# Extracellular vesicles from apoptotic BMSCs ameliorate osteoporosis via transporting regenerative signals

**DOI:** 10.7150/thno.96174

**Published:** 2024-06-03

**Authors:** Maojiao Li, Qi Tang, Chengcheng Liao, Zhuo Wang, Siyuan Zhang, Qingqing Liang, Cheng Liang, Xiaodong Liu, Jingyi Zhang, Weidong Tian, Li Liao

**Affiliations:** 1 State Key Laboratory of Oral Diseases & National Clinical Research Center for Oral Diseases & Engineering Research Center of Oral Translational Medicine, Ministry of Education & National Engineering Laboratory for Oral Regenerative Medicine, West China Hospital of Stomatology, Sichuan University, Sichuan 610041, China.; 2 Chengdu Shiliankangjian Biotechnology Co., Ltd., China.

**Keywords:** mesenchymal stem cell apoptosis, apoptotic cell-derived extracellular vesicles, cytotherapy, osteoporosis, Ras pathway

## Abstract

**Rationale:** Mesenchymal stromal cells (MSCs) are considered a promising resource for cell therapy, exhibiting efficacy in ameliorating diverse bone diseases. However, most MSCs undergo apoptosis shortly after transplantation and produce apoptotic extracellular vesicles (ApoEVs). This study aims to clarify the potential role of ApoEVs from apoptotic MSCs in ameliorating osteoporosis and molecular mechanism.

**Methods:** In this study, Dio-labeled bone marrow mesenchymal stem cells (BMSCs) were injected into mice to track BMSCs apoptosis and ApoEVs production. ApoEVs were isolated from BMSCs after inducing apoptosis, the morphology, size distribution, marker proteins expression of ApoEVs were characterized. Protein mass spectrometry analysis revealed functional differences in proteins between ApoEVs and BMSCs. BMSCs were adopted to test the cellular response to ApoEVs. Ovariectomy mice were used to further compare the ability of ApoEVs in promoting bone formation. SiRNA and lentivirus were used for gain and loss-of-function assay.

**Results:** The results showed that BMSCs underwent apoptosis within 2 days after being injected into mice and produce a substantial quantity of ApoEVs. Proteomic analysis revealed that ApoEVs carried a diverse functional array of proteins, and easily traversed the circulation to reach the bone. After being phagocytized by endogenous BMSCs, ApoEVs efficiently promoted the proliferation, migration, and osteogenic differentiation of BMSCs. In an osteoporosis mouse model, treatment of ApoEVs alleviated bone loss and promoted bone formation. Mechanistically, ApoEVs carried Ras protein and activated the Ras/Raf1/Mek/Erk pathway to promote osteogenesis and bone formation *in vitro* and *in vivo*.

**Conclusion:** Given that BMSC-derived ApoEVs are high-yield and easily obtained, our data underscore the substantive role of ApoEVs from dying BMSCs to treat bone loss, presenting broad implications for cell-free therapeutic modalities.

## Introduction

Osteoporosis (OP) is a chronic and disabling disease that presents with low bone mass, serious microarchitectural destruction of bone tissue, and high risk of fragility fractures, which is highly detrimental to both society and individuals 1. The pathogenesis of OP is mainly thought to be a decrease in bone formation ability and enhanced osteoclast-mediated bone resorption 2. At present, most of the first-line anti-osteoporotic drugs are anti-catabolic agents that inhibit bone resorption. Although anti-catabolic treatment can enhance bone mass, these drugs cannot restore the physiological balance between bone formation and resorption, resulting in low efficiency in preventing non-stress osteoporosis. Therefore, finding novel osteoanabolic agents that can enhance bone formation is becoming a critical issue in the treatment of osteoporosis.

Mesenchymal stem cells (MSCs) have been widely used in cytotherapeutic in tissue regeneration and disease treatment, given their self-renewal, multilineage differentiation, and immunoregulatory properties. Hundreds of clinical trials applying MSC transplantation (MSCT) to treat conditions such as ischemic heart failure, osteonecrosis, osteoarthritis, and more 3-6 have been initiated or accomplished. However, despite the therapeutic efficacy of mesenchymal stem cells, their therapeutic applications still face significant costs and challenges as they require strict monitoring of manufacturing, processing, and storage to ensure optimal survival and efficacy after transplantation 7, 8. Emerging evidence has revealed that the majority of transplanted MSCs maintain viability for only a brief duration, typically a few days, after which they undergo apoptosis either in the host circulation or in engrafted tissues 9. The beneficial outcomes of stem cell therapy may be attributed to nonviable cells. Apoptosis plays a crucial role in the maintenance of tissue homeostasis 10, 11. Apoptotic cells can provide specific signals to promote tissue development or maintain tissue homeostasis via a process of efferocytosis. During apoptosis, a large number of apoptotic cells derived extracellular vesicles (ApoEVs) are produced. ApoEVs encapsulate the versatile components of dead cells including proteins (e.g., proteins from the nucleus, mitochondria, and plasma membrane), lipids, and nucleic acids (e.g., mRNA, long non-coding RNA, rRNA, miRNA, or fragments of these RNA molecules) 12-14, thereby acting as new bioactive carriers in balancing death and regeneration 15, 16. In a previous study, we examined the characteristics and specific markers of MSC-derived ApoEVs and found that ApoEVs can promote bone regeneration by local injection 9. In another study, we demonstrated that ApoEVs promote high-quality skin wound healing, compared with other type of vesicles 17. MSC-derived ApoEVs are found to decrease myocardial infarction 18, ameliorate inflammation and joint erosion in a mouse model of arthritis 19. While ApoEVs have been used to promote bone regeneration, it is still unclear whether the therapeutic effects of intravenously administered MSCs on osteoporosis is mediated by ApoEVs from the apoptotic MSCs.

In this study, we hypothesize that ApoEVs, serving as a novel mechanism in MSCT, play a role in osteoporosis treatment after BMSCs transplantation. Our studies have traced the post-transplantation fate of BMSCs and observed the generation and distribution of ApoEVs. We found that ApoEVs can reach bone tissue and be taken up by endogenous BMSCs. ApoEVs promoted the proliferation and osteogenic differentiation of BMSCs, thereby stimulating bone formation in OVX mice. Through the analysis of differentially expressed proteins in ApoEVs from BMSCs, we identified the ApoEVs promoted bone formation by transporting Ras to activate the Ras/Raf1/Mek/Erk pathway in BMSCs. Overall, our data provide an important theoretical basis for novel clinical applications of ApoEVs.

## Results

### BMSCs underwent apoptosis and generated ApoEVs post-transplantation

To verify the fate of BMSCs after transplantation, an *In Vivo* Imaging System (IVIS) was used (Figure S1A). The fluorescence signals from main organs (heart, lung, liver, spleen, brain, legs, and mandible) were examined at 1 h, 6 h, 24 h, and 48 h after transplantation of Dio-labeled BMSCs. The results demonstrated that the Dio-BMSCs migrated to various locations, including the skeleton, lung, liver, kidneys, adipose tissue, among others (Figure S1B). Quantitative analysis of fluorescence intensity showed that Dio in the femur/tibia or mandible reaches its peak at 24 h, significantly decreasing at 48 h (Figure S1C). To verify the time required for apoptosis of BMSCs *in vivo* after transplantation, histologically analyzed the survival of ZsGreen-BMSCs in various organs (heart, liver, spleen, lung, kidney) on the 2nd and 5th day of tail vein injection, respectively. ZsGreen-BMSCs could be detected in heart, liver, and kidney 2 days after tail vein injection, but were almost undetectable on the 5th day (Figure S1D), suggesting that the majority of BMSCs underwent apoptosis. Next, we sought to ascertain whether transplanted BMSCs underwent apoptosis during the early stage of bone repair. GFP^+^ BMSCs sheets were transplanted into the femoral defect area of normal C57BL/6J mice and harvested for analysis 2nd after transplantation (Figure S1E). TUNEL staining showed that a notable decrease in the number of GFP^+^ BMSCs and a concomitant increase in apoptotic cells post-transplantation (Figure S1F).

**Figure 1:**
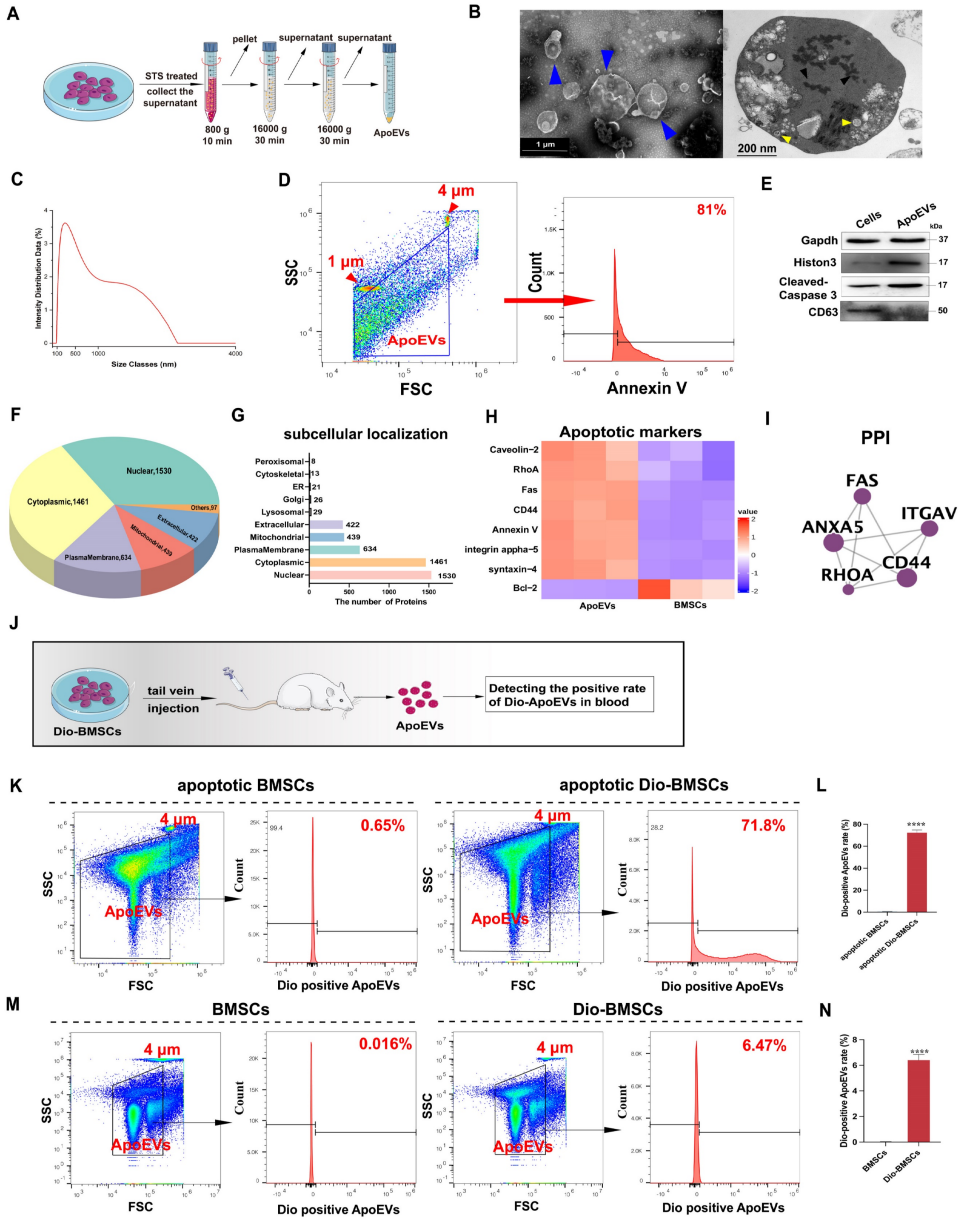
** BMSCs underwent apoptosis post-transplantation and produced ApoEVs.** (**A**) Schematic illustration of ApoEVs isolation via differential centrifugation. Morphology (**B**), diameter distribution (**C**), ApoEVs marker analysis (**D**), and expresssion of Gapdh, Histon 3, Caspase 3, and CD63 (**E**) in the identified ApoEVs. Blue arrows pointed out the ApoEVs. The black arrows denote nucleus fragments, and the yellow arrows indicate the autophagosomes. Scale bar: 1 μm (left); 200nm (Right). (**F-G**) Pie and bar charts showed subcellular localizations of total proteins in ApoEVs based on the proteomic profile. (**H**) Clustering heatmap o illustrates apoptotic markers in ApoEVs. (**I**) PPI network analysis of ubiquitous and specific ApoEVs markers. (**J**) Schematic diagram of the experimental design for tracking ApoEVs production post-BMSCs transplantation. (**K-L**) FCM analysis of the Dio-positive ApoEVs ratio from Dio-labeled BMSCs after apoptosis induction by STS. (**M-N**) FCM showed the Dio-positive ApoEVs ratio 48 h after tail vein injection of Dio-labeled BMSCs *in vivo*. Data are means ± SEMs. Statistical significance was determined by two-tailed paired t-test in (L, N). Not significant (ns) = P > 0.05, *P < 0.05, **P < 0.01, ***P < 0.001, ****P < 0.0001.

We next investigated whether BMSCs generated ApoEVs after apoptosis. First, we applied an *in vitro* apoptosis model of mouse BMSCs (m-BMSCs) using Staurosporine (STS) treatment. ApoEVs were isolated utilizing an optimized gradient centrifugation protocol (Figure 1A) 20. Transmission Electron Microscopy (TEM) analysis confirmed the presence of round-shaped vesicles surrounded by a bilayer membrane. Apoptotic bodies (ApoBDs), defined as vesicles larger than 1 μm, and apoptotic cell-derived microvesicles (ApoMVs), defined as those smaller than 1 μm 15, were observed. Ultrathin sections of ApoEVs imaged by TEM displayed nucleus fragments, cytoplasmic organelles, and autophagosomes, which were also presented in apoptotic cells (Figure 1B). The Dynamic Light Scattering (DLS) showed that the particle size distribution chart and the diameter range of ApoEVs is from 50 to 5000 nm after the separation process (Figure 1C). NTA was used to detect the diameter range and nanoparticles of small ApoMVs, showing that 4.6×10^9^ particles/mL ApoEVs was generated from 1×10^6^ BMSCs (Figure S2A). We detected the ratio of ApoEVs positive for Annexin V was up to 81% (Figure 1D). Western blot analysis showed that ApoEVs contained specific markers of ApoEVs, including histone 3, and cleaved-caspase 3, while lacking the exosome markers (CD63) (Figure 1E). BCA detected the protein mass of ApoEVs from BMSCs, showing that 1×10^6^ BMSCs can produce approximately 450 μg ApoEVs at 4 h after STS inducing apoptosis (Figure S2B). High-performance liquid chromatography-mass spectrometry (HPLC-MS/MS) showed no STS residue in the extracted ApoEVs (Figure S2C), proving the security of this extraction method. Next, to identify the specific proteomic features of ApoEVs, we extracted proteins from ApoEVs and performed LC-MS/MS analysis. The subcellular localizations of total expressed proteins were primarily in the nucleus, cytosol, plasma membrane, mitochondria, extracellular space, and lysosome (Figure 1F-G). Proteomics analysis revealed a significant upregulation of several key apoptotic markers in ApoEVs, while anti-apoptotic protein (Bcl-2) was downregulated, confirming the identity of ApoEVs (Figure 1H-I).

Then, we used Flow Cytometry (FCM) to verify whether BMSCs post-transplantation can produce ApoEVs *in vivo* (Figure 1J). First, BMSCs were labeled with Dio before induction of apoptosis *in vitro*. FCM showed that 71.8% of ApoEVs generated by apoptotic Dio-labeled BMSCs were Dio-positive, indicating the viability of this method for labeling ApoEVs (Figure 1K-L). Subsequently, Dio-labeled BMSCs were injected into the tail vein of mice, and ApoEVs were collected from blood to detect the positive Dio-ApoEVs rate. The Dio-positive rate in ApoEVs isolated from blood was detected by FCM after Dio-BMSCs were injected at 1, 2, and 5 days (Figure 1M-N, Figure S3A-B). The results showed that the rate of Dio-positive ApoEVs gradually increased within 2 days of BMSCs injection, then gradually decreased, and was basically undetectable after the 5 days of injection, suggesting BMSCs undergo apoptosis and generate ApoEVs post-transplantation.

### ApoEVs can migrate into bone via circulation and be uptaken by local BMSCs

**Figure 2:**
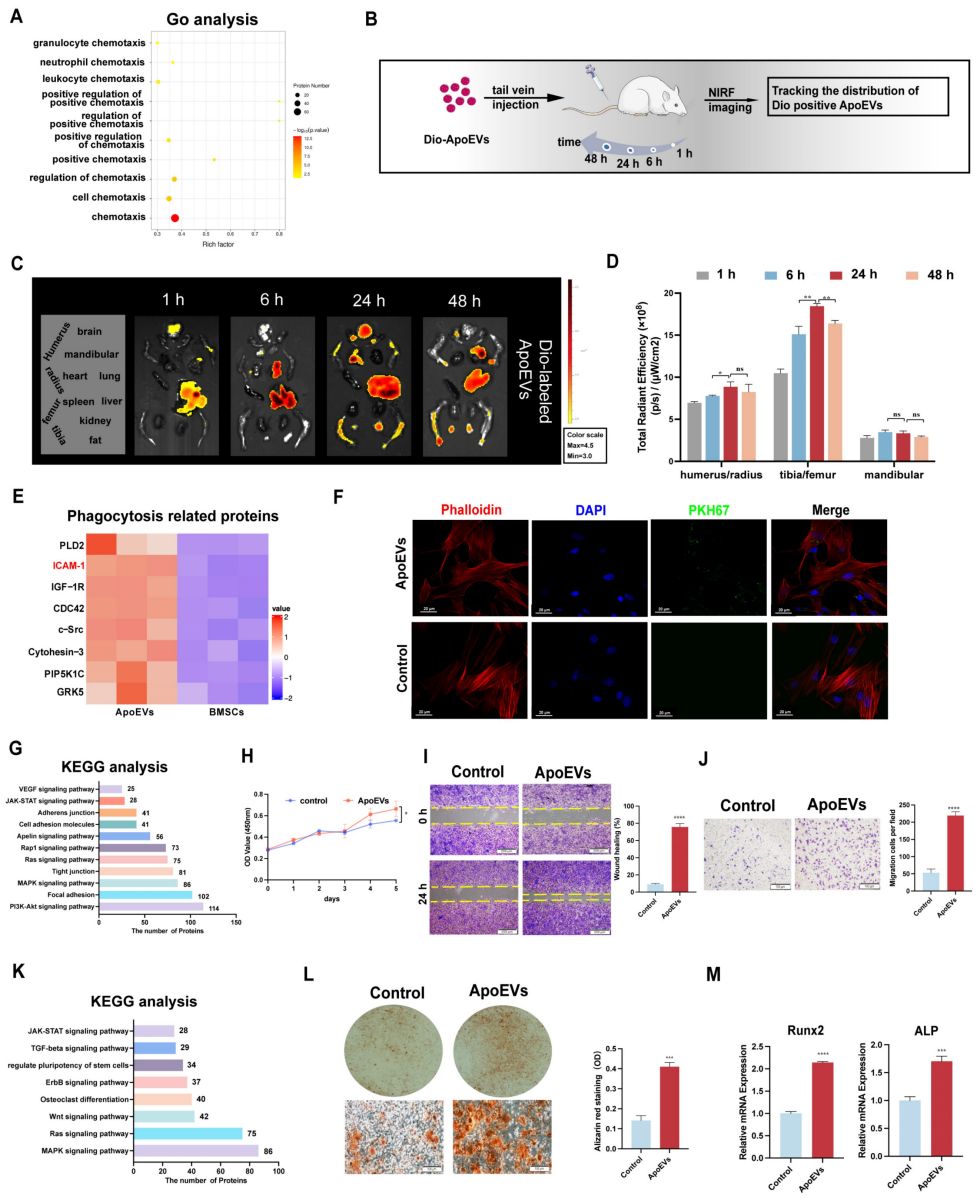
** ApoEVs reach bone and enhance the regeneration potential of BMSCs.** (**A**) Go analysis revealed the association of ApoEVs with 'cell chemotaxis-related annotations'. (**B**) Schematic diagram of the experimental design for *in vivo* tracking of ApoEVs distribution. (**C-D**) Fluorescence intensity of major organs at 1h, 6h, 24h, and 48h post-injection of Dio-labeled ApoEVs. (**E**) Clustering heatmap of phagocytosis-related proteins in ApoEVs. (**F**) PKH67-labeled ApoEVs engulfed by m-BMSCs at 24 h co-cultured. Scale bar: 20 μm. (**G**) KEGG analysis highlights proliferation and adhesion-related pathways in ApoEVs. (**H**) The impact of ApoEVs on BMMSC proliferation measured by the CCK-8 assay. (**I-J**) Effect of ApoEVs on BMMSC migration and invasion measured by scratch healing assay and transwell assay. Scale bar: 200 μm (I), 100 μm (J). (**K**) KEGG analysis indicates osteogenic differentiation- related pathways in ApoEVs. (**L**) Alizarin red staining and qRT-PCR analysis of Runx2 and ALP (**M**) in BMSCs treated with ApoEVs under osteogenic induction. Scale bar: 100 μm. Data are means ± SEMs. Statistical significance was determined by two-tailed paired t-test in (D, H, I, J, L, M). Not significant (ns) = P > 0.05, *P < 0.05, **P < 0.01, ***P < 0.001, ****P < 0.0001.

According to the results of the protein mass spectrometer, we observed ApoEVs expressed numerous chemotaxis factors (Figure 2A). To verify whether ApoEVs could migrate toward specific tissue via circulation, Dio-labelled ApoEVs were injected into mice and their distribution was detected by the NIFR imaging system (Figure 2B). The fluorescence signals of the main organs (heart, lung, liver, spleen, brain, limbs, and mandible) separated from the mice were examined at 1 h, 6 h, 24 h, and 48 h (Figure 2C). It was observed that ApoEVs accumulated in all bone tissues (humerus/radius, femurs/tibias, and mandibular). Notably, while the signaling in other organs (liver, lung) showed an obvious attenuation 24 h after injection (Figure S4A), the fluorescence signal in the bone tissue significantly aggregated 24 h after injection and was maintained without an obvious decrease after 48 h (Figure 2D), indicating that ApoEVs can reach the bone via circulation.

Furthermore, some phagocytosis-related proteins, especially the 'eat-me' signal ICAM-1, were expressed in ApoEVs (Figure 2E). Building upon this observation, we proceeded to elucidate that ApoEVs can be engulfed by BMSCs in the microenvironment. After co-culturing with ApoEVs for 24 h, BMSCs could successfully uptake exogenous PKH67-labeled ApoEVs (Figure 2F). These findings suggest that ApoEVs derived from BMSCs can migrate to bone tissue and be engulfed by endogenous BMSCs.

### ApoEVs promote the ability of proliferation, migration, and osteogenic differentiation of BMSCs

Next, we aimed to investigate whether the engulfment of ApoEVs can trigger functional responses in BMSCs. KEGG pathway analysis revealed that proteins of ApoEVs were associated with proliferation-related pathways, such as 'PI3K-Akt signaling pathway' and 'MAPK signaling pathway'; as well as some adhesion-related annotations, such as 'tight junction' and 'cell adhesion pathway' (Figure 2G). The growth curves determined by the CCK-8 assay showed that 10 μg/mL ApoEVs promoted m-BMSCs proliferation compared with the control group (Figure 2H, Figure S5A). We used the scratch healing assay and Boyden chamber assay to verify the effect of ApoEVs on the migration of BMSCs. The scratch healing assay showed that ApoEVs accelerated the movement of m-BMSCs to the scratch area (Figure 2I). The Boyden chamber assay showed that ApoEVs accelerated the migration of m-BMSCs from the upper chamber to the lower chamber (Figure 2J).

KEGG pathway analysis revealed that proteins of ApoEVs were associated with osteogenesis-related pathways, such as the 'Wnt signaling pathway' and 'regulate pluripotent of stem cells' (Figure 2K). To verify the osteogenic potency of ApoEVs on BMSCs, m-BMSCs were co-cultured with ApoEVs during osteogenic induction. Alizarin red staining showed that ApoEVs enhanced the capacities of m-BMSCs for osteogenic differentiation (Figure 2L). We confirmed that the level of osteogenic Runx2 and ALP in recipient BMSCs were upregulated after exposure to ApoEVs (Figure 2M).

To confirm that the effect of ApoEVs was not species-specific, we also repeated the above experiment using BMSCs and ApoEVs derived from rats. The CCK-8 assay results (Figure S5B, S6A) showed that 10 μg/mL ApoEVs from rat BMSCs (r-BMSCs) also promoted the growth and proliferation, migration (Figure S6B-C), and osteogenic differentiation (Figure S6D-F) of r-BMSCs. These results were consistent with the effect of mouse ApoEVs on m-BMSCs.

Taken together, these results indicated that ApoEVs being engulfed by BMSCs can promote their proliferation, migration, and osteogenic differentiation.

### ApoEVs protect against bone loss in osteoporosis

**Figure 3:**
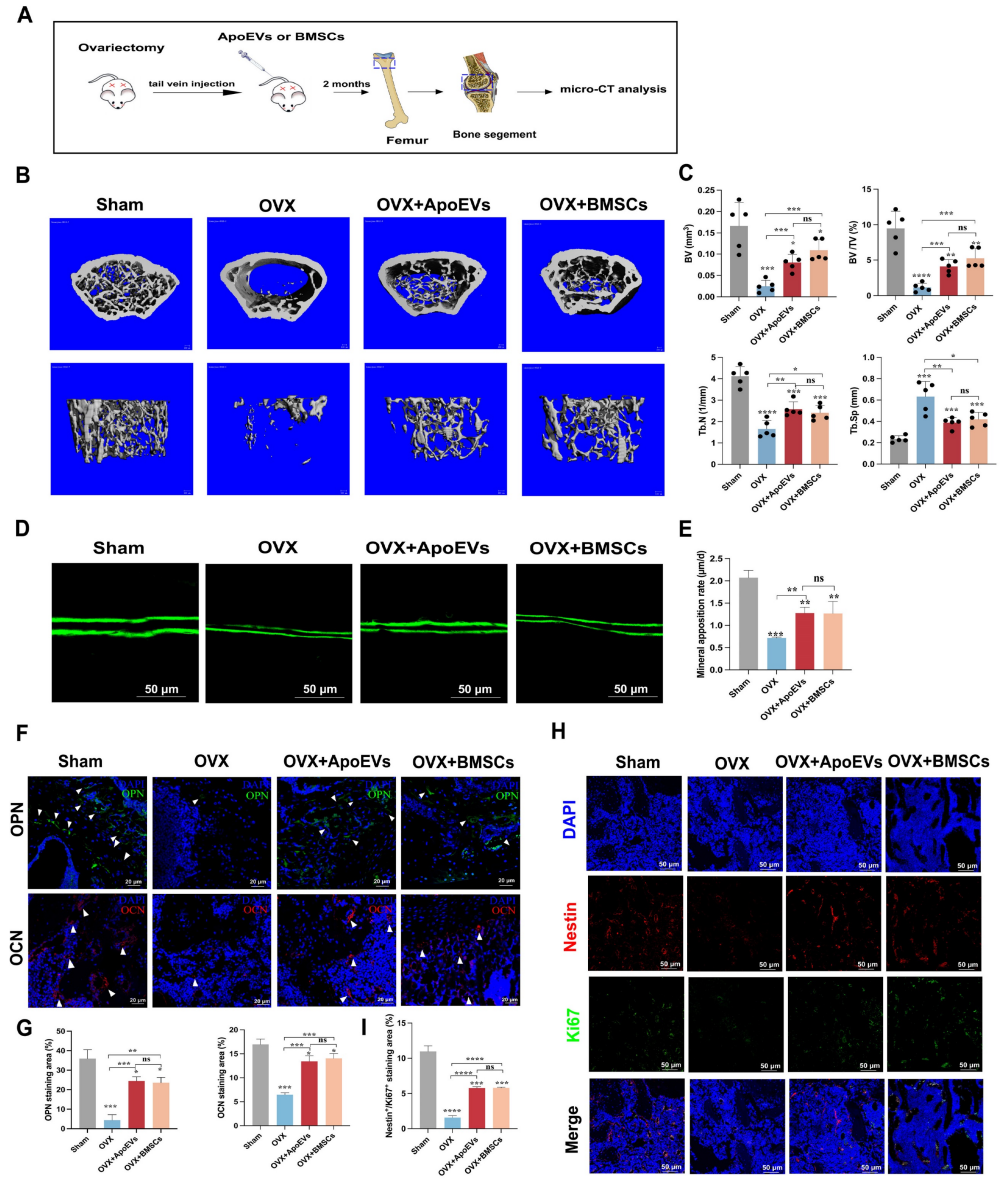
** ApoEVs protect against bone loss in osteoporotic mice.** (**A**) Schematic view of the experimental procedure. BMSCs and ApoEVs were administered into OVX mice weekly for two months. (**B**) Three-dimensional reconstruction images of μCT illustrating bone microarchitecture in the distal femur. (**C**) Quantitative analysis of BV, BV/TV, Tb.N, and Tb.Sp based on μCT examination. n=5 biologically independent animals per group. (**D-E**) Calcein double labeling depicting bone formation rate per bone surface values. (**F-G**) Immunofluorescence staining visualizing the expression of OPN and OCN. (**H-I**) IF was used to detect and analyze the proliferation (green) of Nestin+ BMSCs (Red) after ApoEVs and BMSCs injected into OVX mice. Scale bar: 50 μm. Data are means ± SEMs. Statistical significance was determined by two-tailed paired t-test in (C, E, G, I), compared with Sham group. Not significant (ns) = P > 0.05, *P < 0.05, **P < 0.01, ***P < 0.001, ****P < 0.0001.

Considering ApoEVs promote the regenerative potency of BMSCs *in vitro*, we compared the therapeutical effect of ApoEVs and BMSCs on osteoporosis in a mouse model. ApoEVs and BMSCs were injected via the tail vein into OVX mice once a week for 2 months. We performed microCT detection of the distal femur of OVX mouse models after treatment (Figure 3A). The 3D reconstruction of the bone segment and parameters of trabecular bone showed that the treatment of ApoEVs markedly enhanced the bone volume and protected the microarchitecture of trabecular bone in OVX mice (Figure 3B-C). Moreover, the double calcein labeling assay and immunofluorescence (IF) staining of osteopontin (OPN) and osteocalcin (OCN) confirmed that treatment with ApoEVs increased the formation rate of new bone compared with OVX mice (Figure 3D-G). Notably, the therapeutic effect of ApoEVs was similar to BMSCs administration (Figure 3B-G). IF staining showed ApoEVs treatment also promoted endogenous Nestin^+^ BMSCs proliferation (Figure 3H-I). Taken together, these data support our conclusion that ApoEVs, like the BMSCs therapy, could facilitate the bone formation of trabecular bone in osteoporosis mice.

The balance between bone formation by osteoblasts and bone resorption by osteoclasts is important for maintaining bone homeostasis. We further analyzed the role of ApoEVs on osteoclast formation *in vitro* and *in vivo*. Tartrate-resistant acid phosphatase (TRAP) staining showed fewer osteoclasts in the osteoclastic medium (OCM) + ApoEVs group than in the OCM group (Figure S7A-B). *In vivo* analysis, TRAP staining showed fewer TRAP-positive cells in the femur of OVX mice injected with ApoEVs (Figure S7C-D), suggesting that ApoEVs can also inhibit osteoclast formation and bone resorption.

### ApoEVs enhance the osteogenic activity of BMSCs by transporting Ras protein

**Figure 4:**
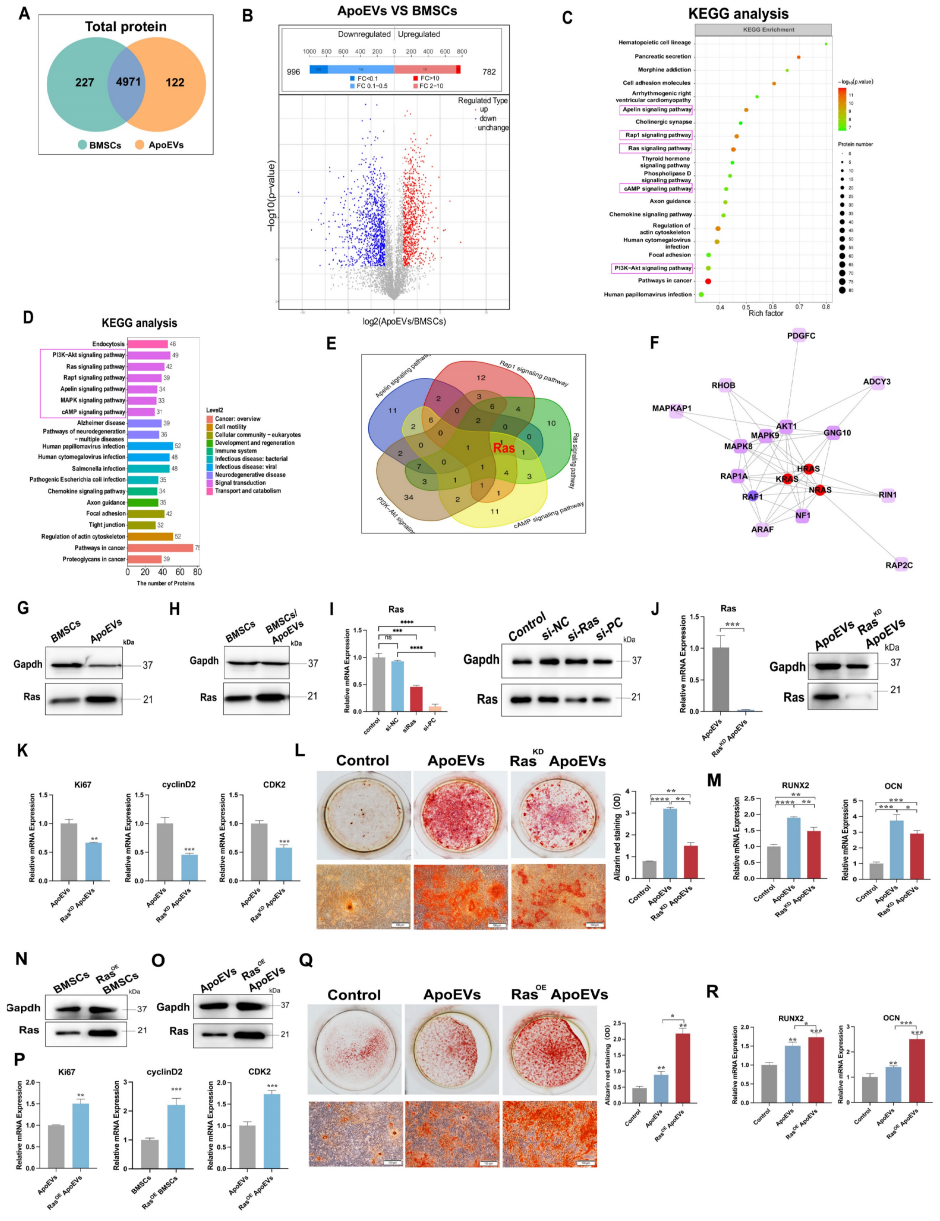
** ApoEVs enhance the proliferation and osteogenic differentiation of BMSCs via Ras protein transport.** (**A**) Venn diagram illustrating the numbers of unique and overlapping proteins between ApoEVs and BMSCs. (**B**) Volcano plots depicting significantly upregulated (red dots) and downregulated (blue dots) proteins in ApoEVs compared to BMSCs. Differentially expressed proteins (DEPs) were determined by fold change ≥ 2 and adjusted p-value < 0.05. (**C-D**) KEGG enrichment analysis of significantly upregulated pathways in ApoEVs compared to BMSCs. (**E**) Venn diagram showing the involvement of Ras in most of these selected pathways (F) PPI network analysis of Ras and Ras-related proteins. (**G**) Western blot analysis indicating high Ras expression in ApoEVs and upregulation of Ras expression in BMSCs following ApoEV treatment (**H**). (**I**) SiRNA-mediated knockdown of Ras expression in BMSCs, confirmed by QRT-PCR and western blot. (**J**) ApoEVs isolated from these BMSCs exhibited low Ras expression. (**K**) QRT-PCR demonstrating reduced proliferation-promoting ability of Ras^KD^ ApoEVs in BMSCs. (**L**) ARS and qRT-PCR analysis of Runx2 and OCN (**M**) in BMSCs treated with ApoEVs or Ras^KD^ ApoEVs under osteogenic induction. Scale bar: 100 μm. (**N**) Western blot confirming successful transfection of Ras overexpressed lentivirus into BMSCs and high Ras expression in ApoEVs derived from these BMSCs (**O**). n=3 biologically independent cells per group. (**P**) QRT-PCR revealing enhanced proliferation-promoting ability of Ras^OE^ ApoEVs in BMSCs. (**Q**) ARS and qRT-PCR analysis of Runx2 and OCN (**R**) in BMSCs treated with ApoEVs or Ras^OE^ ApoEVs under osteogenic induction. Scale bar: 100 μm. Data are means ± SEMs. Statistical significance was determined by two-tailed paired t-test in (I, J, K, L, M, P, Q, R). Not significant (ns) = P > 0.05, *P < 0.05, **P < 0.01, ***P < 0.001, ****P < 0.0001.

To identify the specific bioactive factors of ApoEVs, we compared the proteins of ApoEVs and parental BMSCs using LC-MS/MS analysis. Among the 5320 proteins that were identified (Figure 4A), 782 proteins were significantly upregulated in ApoEVs (Figure 4B). We then focused on these differentially expressed proteins (DEPs) and carried out KEGG pathway analysis. The results showed that these proteins were closely associated with six signal transduction-related pathways, including 'PI3K-Akt signaling pathway', 'Ras signaling pathway', 'Rap1 signaling pathway', 'Apelin signaling pathway', 'MAPK signaling pathway', and 'cAMP signaling pathway' (Figure 4C-D). Specifically, we found that 'Ras' is collectively expressed in these pathways (Figure 4E). Elaborate network analysis of Ras by PPI showed that Ras played a central role in the crosstalk of these signaling pathways (Figure 4F). Consistent with the bioinformatic analysis, western blot analysis showed that Ras was highly expressed in ApoEVs compared to BMSCs (Figure 4G). Besides, ApoEVs treatment also upregulated the expression of Ras in BMSCs (Figure 4H). Therefore, we chose Ras as the candidate for the following experiments.

The Ras protein acts as a molecular switch, connecting extracellular signals from cell surface receptors to intracellular pathways to regulate cell proliferation, differentiation, aging, and death 21. Based on this knowledge, we verify whether Ras plays a crucial role in ApoEVs regulating BMSCs through gain and loss-of-function assay. We successfully used siRNA to knock down Ras (Figure 4I) and lentivirus to overexpress Ras in BMSCs (Figure S8, Figure 4N), respectively.

As expected, ApoEVs from BMSCs knocked down of Ras (Ras^KD^ ApoEVs) had a low expression of Ras (Figure 4J). ApoEVs generated from Ras-overexpressed BMSCs (Ras^OE^ ApoEVs) had a high expression of Ras (Figure 4O). Then, we examined whether knocking down or overexpression Ras could affect ApoEVs-induced proliferation and osteogenic differentiation. After knocking down Ras, ApoEVs were less effective in inducing the expression of proliferation-related genes (Ki67, cyclin D2, and CDK2) in BMSCs after treatment (Figure 4K), while overexpression of Ras in ApoEVs resulted in high expression of these genes in BMSCs (Figure 4P), indicating that Ras promotes the proliferation of BMSCs. Besides, downregulation of Ras in ApoEVs significantly inhibited the ability of ApoEVs to promote osteogenic differentiation in BMSCs (Figure 4L-M), while overexpression of Ras can expand these abilities (Figure 4Q-R). These results suggest that Ras, as a key protein, is involved in the regulation of ApoEVs on the proliferation and osteogenic differentiation of BMSCs.

**Figure 5:**
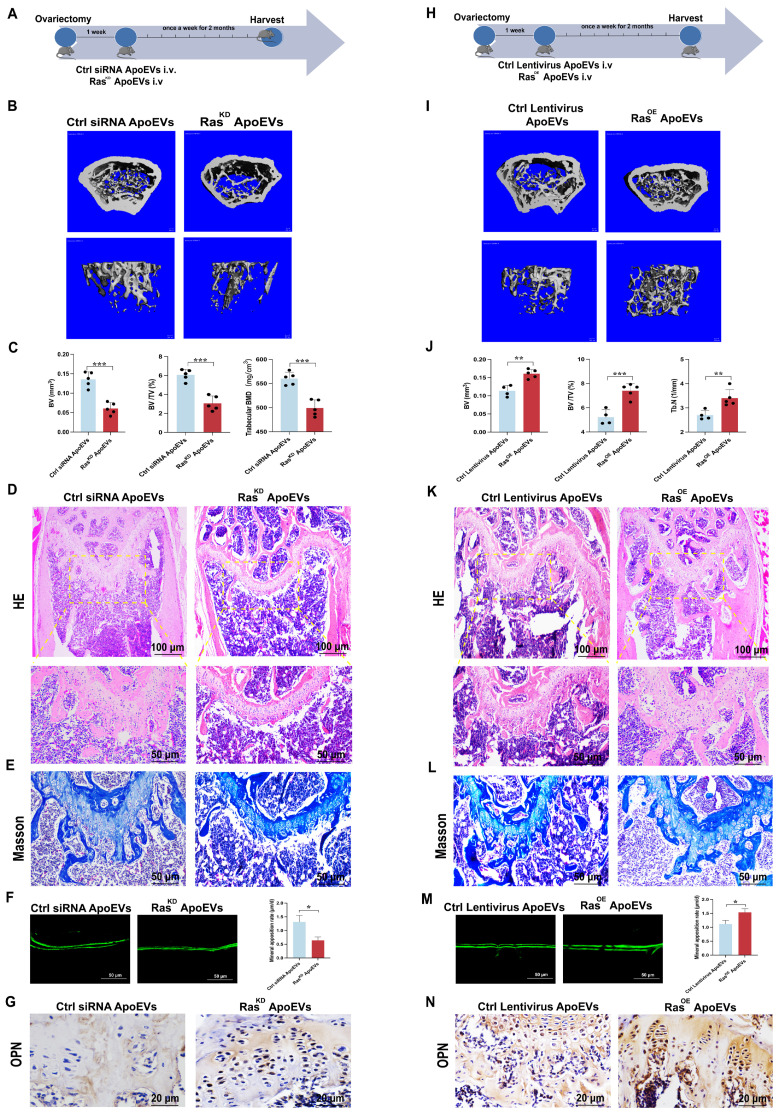
** ApoEVs ameliorate osteoporosis via Ras signal.** (**A**) Schematic view of the experimental procedure. Control siRNA ApoEVs and Ras^KD^ ApoEVs were injected into OVX mice weekly for two months. (**B-C**) Three-dimensional reconstruction images of μCT illustrating bone microarchitecture in the distal femur, and quantitative analysis of BV, BV/TV, and BMD. n=5 biologically independent animals per group. (**D**) HE and (**E**) Masson depicting fewer osteoblast-like cells at the growth plate metaphysis in Ras^KD^ ApoEVs treated mice. Scale bar: 100 μm and 50 μm (D); 50μm (E). (**F**) Calcein double labeling indicating a slower bone formation rate per bone surface in Ras^KD^ ApoEVs group. (**G**) IHC staining visualizing lower expression of OPN in Ras^KD^ ApoEVs group. Scale bar: 20 μm. (**H**) Schematic view of the experimental procedure. Control lentivirus ApoEVs and Ras^OE^ ApoEVs were injected into OVX mice weekly for two months. (**I-J**) Three-dimensional reconstruction images of μCT depicting bone microarchitecture in the distal femur, and quantitative analysis of BV, BV/TV, and Tb.N. n=4 biologically independent animals per group. (**K**) HE and (**L**) Masson staining revealing more osteoblast-like cells at the growth plate metaphysis in Ras^OE^ ApoEVs treated mice. Scale bar: 100 μm and 50 μm (K); 50μm (L). (**M**) Calcein double labeling demonstrating a faster bone formation rate per bone surface in the Ras^OE^ ApoEVs group. (**N**) IHC staining showing lower expression of OPN in Ras^OE^ ApoEVs group. Scale bar: 20 μm. Data are means ± SEMs. Statistical significance was determined by two-tailed paired t-test in (C, F, J, M). Not significant (ns) = P > 0.05, *P < 0.05, **P < 0.01, ***P < 0.001, ****P < 0.0001.

Next, Ras^KD^ ApoEVs or Ras^OE^ ApoEVs were injected into the OVX mice, respectively, to verify the effect of Ras in ApoEVs on ameliorating osteoporosis *in vivo* (Figure 5A, H). Micro-CT demonstrated that Ras^KD^ ApoEVs was less effective in preventing bone loss in OVX mice compared with the control siRNA ApoEVs group (Figure 5B-C), while Ras^OE^ ApoEVs prevented bone loss more effectively than the control ApoEVs (Figure 5I-J). HE staining and Masson's trichrome staining confirmed the results of micro-CT analysis (Figure 5D-E, K-L). The Ras^KD^ ApoEVs groups had a lower rate of new bone formation (Figure 5F), while the Ras^OE^ ApoEVs group had a faster rate of new bone formation (Figure 5M). Immunohistochemistry (IHC) staining revealed that OPN was less expressed in Ras^KD^ ApoEVs-treated OVX mice (Figure 5G), but more expressed in Ras^OE^ ApoEVs-treated OVX mice (Figure 5N). In general, we conclude that Ras is the essential component in ApoEVs that enhances osteogenesis and rescues OVX-induced bone loss.

### ApoEVs activates the Ras/Raf1/Mek/Erk pathway to promote bone formation

**Figure 6:**
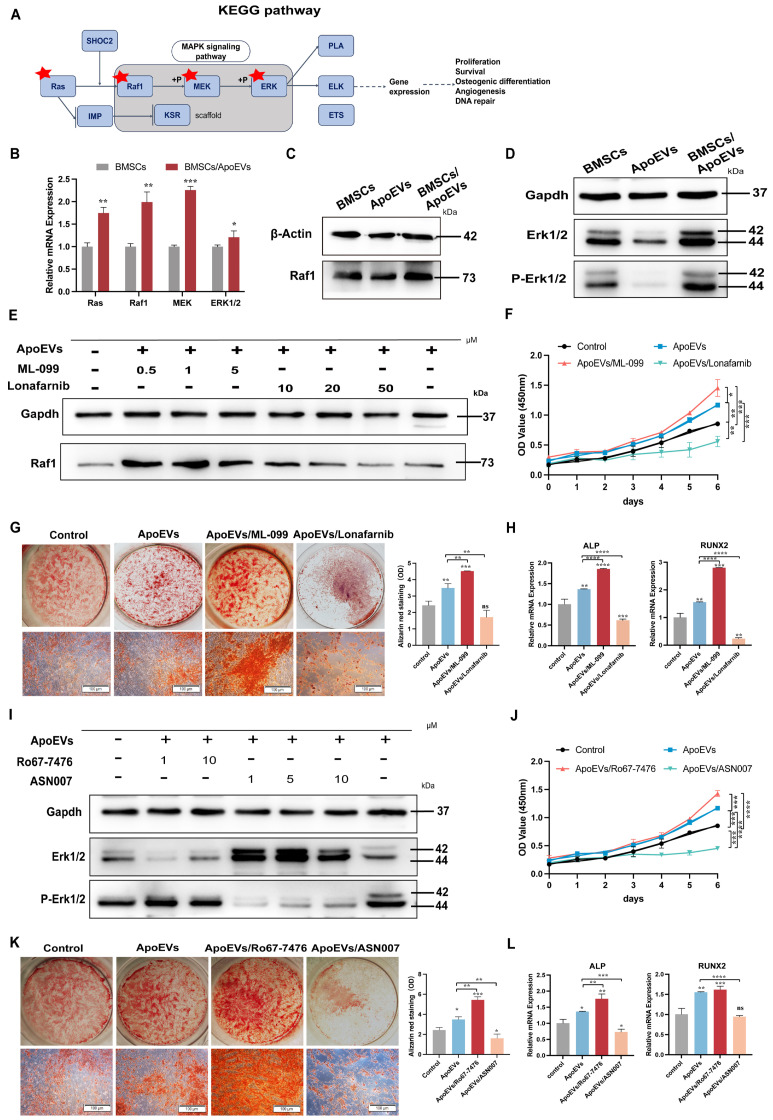
** ApoEVs promoted the proliferation and osteogenic differentiation of BMSCs via the Ras/Raf1/Mek/Erk pathway.** (**A**) KEGG analysis of the Ras pathway. (**B**) QRT-PCR analysis showing the expression of Ras, Raf1, Mek, and Erk in BMSCs after ApoEVs treatment. (**C**) Western blot analysis showing upregulated Raf1 expression and active p-Erk1/2 (**D**) in BMSCs after ApoEVs treatment. (**E**) Western blot analysis indicating Raf1 expression in BMSCs treated with ApoEVs, ML-099, or Lonafarnib. (**F**) CCK-8 assay showing the growth curve of BMSCs treated with ApoEVs, ML-099, or Lonafarnib. (**G**) ARS and qRT-PCR analysis of ALP and RUNX2 (**H**) in BMSCs treated with ApoEVs, ML-099, or Lonafarnib under osteogenic induction. Scale bar: 100 μm. (**I**) Western blot analysis showed the expression of Erk and p-Erk in BMSCs treated with ApoEVs, Ro67-7476, or ASN007. (**J**) CCK-8 assay showing the growth curve of BMSCs treated with ApoEVs, Ro67-7476, or ASN007. (**K**) ARS and qRT-PCR analysis of ALP and RUNX2 (**L**) in BMSCs treated with ApoEVs, ML-099, or Lonafarnib under osteogenic induction. Scale bar: 100 μm. Data are means ± SEMs. Statistical significance was determined by two-tailed paired t-test in (B, F, G, H, J, K, L). Not significant (ns) = P > 0.05, *P < 0.05, **P < 0.01, ***P < 0.001, ****P < 0.0001.

As a downstream signaling pathway of Ras, the Ras/Raf1/Mek/Erk signaling pathway plays a crucial role in cell functions, such as proliferation, differentiation, cell cycle progression, or cell death (Figure 6A) 22-24. In light of this knowledge, we intended to explore whether ApoEVs function via the Ras/Raf1/Mek/Erk pathway in BMSCs. First, ApoEVs treatment markedly upregulated the gene expression profiles of Ras/Raf1/Mek/Erk in BMSCs (Figure 6B). Besides, ApoEVs treatment increased the protein levels of Raf1 (Figure 6C), and phosphorylated-Erk1/2 (p-Erk1/2) in BMSCs (Figure 6D). Further, we promoted the activity or blocked the activity of Ras using Ras agonists (ML-099) and Ras inhibitors (Lonafarnib) to verify whether ApoEVs transfer Ras to activate the expression of Raf1. Western blot analysis showed that 1 μM ML-099 can upregulate the expression of Raf1 in BMSCs after ApoEVs treatment, and 50 μM Lonafarnib can downregulate the expression of Raf1 in BMSCs (Figure 6E). Importantly, activating Ras/Raf1 expanded the ability of ApoEVs to induce proliferation (Figure 6F) and promote osteogenesis of BMSCs (Figure 6G-H), while inhibition of Ras/Raf1 blocked these abilities.

Then, we used p-Erk1/2 agonist (Ro67-7476) and p-Erk1/2 inhibitor (ASN007) to verify whether ApoEVs regulated BMSCs through the Erk signal. 1 μM Ro67-7476 can upregulate the expression of p-Erk1/2 in BMSCs after ApoEVs treatment, and 1 μM ASN007 can downregulate the expression of p-Erk1/2 (Figure 6I). Activating p-Erk1/2 expanded the ability of ApoEVs to induce proliferation (Figure 6J) and promote osteogenesis of BMSCs (Figure 6K-L), while inhibition of p-Erk1/2 blocked these abilities. These results suggest that ApoEVs stimulate the proliferation and osteogenic differentiation of BMSCs via the Ras/Raf1/Mek/Erk pathway.

**Figure 7:**
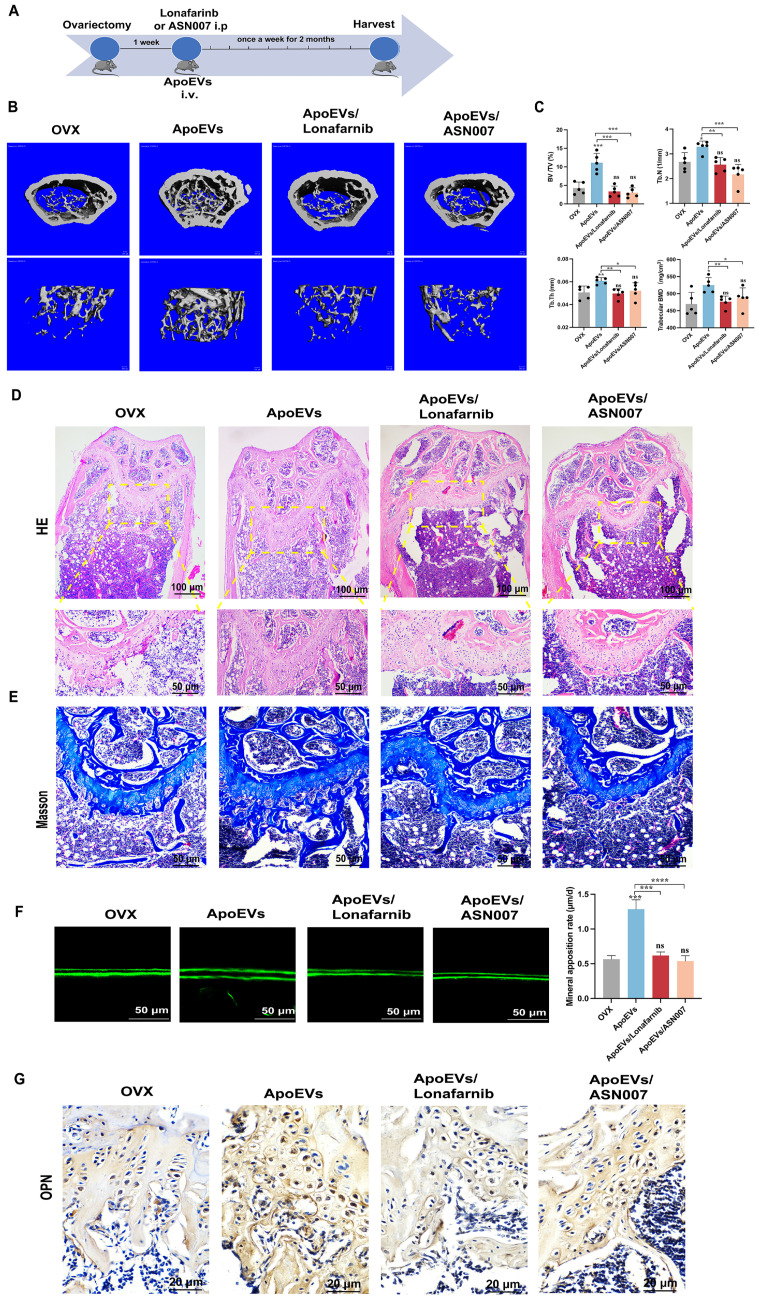
** ApoEVs ameliorate osteoporosis via Ras/Raf1/Mek/Erk pathway.** (**A**) Schematic view of the experimental procedure. ApoEVs, Lonafarnib, or ASN007 were injected into OVX mice weekly for two months. (**B-C**) Three-dimensional reconstruction images of μCT demonstrating bone microarchitecture in the distal femur and quantitative analysis of BV/TV, Tb.N, Tb.Th, and BMD. n=5 biologically independent animals per group. (**D**) HE and (**E**) Masson staining showing the growth plate at the metaphysis. Scale bar: 100 μm and 50 μm (D); 50 μm (E). (**F**) Calcein double labeling showing the bone formation rate per bone surface values. Scale bar: 50 μm (**G**) IHC staining visualized the expression of OPN. Scale bar: 20 μm. Data are means ± SEMs. Statistical significance was determined by two-tailed paired t-test in (C, F), which were compared with OVX group. Not significant (ns) = P > 0.05, *P < 0.05, **P < 0.01, ***P < 0.001, ****P < 0.0001.

**Figure 8:**
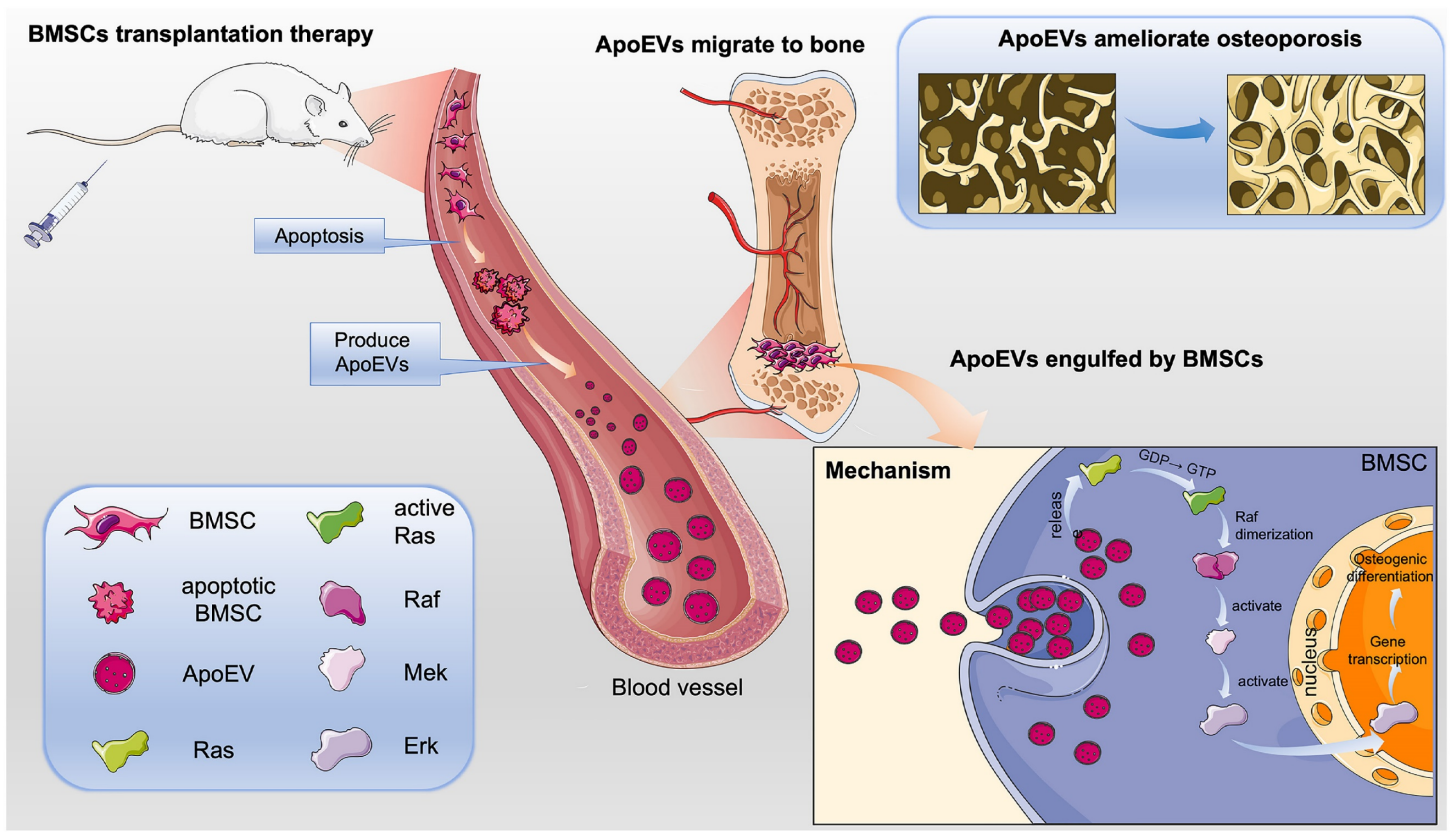
** Schematic illustration of the mechanism of BMSCs transplantation in the treatment of osteoporosis.** BMSCs undergo apoptosis post-transplantation, leading to the secretion of abundant ApoEVs. ApoEVs migrate to bone tissues and are engulfed by endogenous BMSCs, thereby promoting the ability of proliferation and osteogenic differentiation via activating the Ras/Raf1/Mek/Erk pathway. Additionally, ApoEVs can ameliorate osteoporosis and promote bone formation via the Ras/Raf1/Mek/Erk pathway. This suggests a novel mechanism for 'cell-free' stem cell therapy in the treatment of osteoporosis.

To verify the effect of ApoEVs through the Ras/Raf1/Mek/Erk pathway *in vivo*, Lonafarnib and ASN007 were injected into the OVX mice, respectively, after ApoEVs tail vein injection (Figure 7A). The 3D reconstruction of the bone segment showed that the treatment of either Lonafarnib or ASN007 abolished the effect of ApoEVs to enhance the bone volume and protect the microarchitecture of trabecular bone in OVX mice (Figure 7B-C). HE staining and Masson's trichrome staining confirmed that the treatment of Lonafarnib and ASN007 blocked the therapeutic effect of ApoEVs in OVX mice (Figure 7D-E). Moreover, Lonafarnib and ASN007 weakened the ability of ApoEVs on the formation rate of new bone (Figure 7F) and suppressed the expression of OPN in the trabecular bone of OVX mice received ApoEVs treatment (Figure 7G). Taken together, these results support our conclusion that ApoEVs from apoptotic BMSCs post-transplantation facilitate bone formation in osteoporosis mice via the Ras/Raf1/Mek/Erk pathway (Figure 8).

## Discussion

The apoptosis of MSCs after transplantation has been indirectly detected through various modalities such as cryo-imaging 25 and microarray analysis 26. In this study, BMSCs were labeled with a membrane-bound dye (Dio), a method facilitating the precise tracking of cells and cellular products. The majority of Dio-labeled BMSCs was not detected 48 h after transplantation, suggesting robust apoptosis of the transplanted BMSCs. Moreover, highly sensitive FCM-based approaches detected abundant ApoEVs after the administration of Dio-labeled BMSCs, offering evidentiary support for the apoptosis of BMSCs in the circulatory system.

Previous studies have suggested that apoptosis is closely involved in bone formation. For example, apoptosis-deficient mice exhibit poorer osteogenic ability, whereas the stimulation of apoptosis significantly alleviates osteoporosis. Some inflammatory microenvironments differentially modulate MSC function by inducing apoptosis 27. Here, the animal study showed that the treatment of ApoEVs performed a similar therapeutic effect as the treatment of BMSCs. Although we could not exclude the likelihood of a minute population of surviving BMSCs exerting discernible effects, these results suggest that the apoptotic MSCs, other than the viable MSCs, are essential for BMSCs cytotherapy.

By analyzing the protein components of ApoEVs, it was found that ApoEVs revealed a notable enrichment in chemotactic-related proteins. Tracking Dio-labeled ApoEVs demonstrated a pronounced accumulation in the liver initially, followed by a gradual recruitment into bone tissue. In the OVX model, the bone serves as a homing niche and activation site, which ApoEVs must traffic through circulation and reach it. This aligns with the findings of Liu *et al.*, who found that ApoEVs can reach to the femur in multiple myeloma mice 28. Similarly, Wang, J. *et al.* reported ApoEVs reaching to the jawbone to alleviate Pg-LPS-induced inflammatory responses of macrophages 29. Since BMSCs cytotherapy has been applied to a number of diseases in the liver, kidney, heart, skeleton, pancreas, *et al.* The following experiments are necessary to extensively evaluate the therapeutic effect of ApoEVs for other diseases, which will deepen our understanding of the mechanism of MSC cytotherapy.

Extracellular vesicles include exosomes, microvesicles, and ApoEVs 30. As a subset of extracellular vesicles, ApoEVs have some different properties from the other two types of vesicles 31. Larger volumes and a wider diameter distribution made ApoEVs carrying more bioactive contents 32. ApoEVs are derived from apoptotic cell disassembly, more like a natural loading system, in which the contents from progenitor cells are actively sorted into the ApoEVs. Notably, the ApoEVs we used in this article include large ApoBDs and small ApoMVs. Different kinds of ApoEVs may carry different bioactive components to perform different functions. More research is needed to identify the subgroups that play key roles in ApoEVs. Besides, the components of ApoEVs strongly depend on the identity of their parental cells 33. The comparison of bioinformatics analysis results also shows that the content of ApoEVs from different cells and tissues is not completely the same 34, 35. Thus, ApoEVs derived from diverse cell types or tissues contain different cargoes and exhibit distinct functions.

ApoEVs serve as carriers inheriting bioactive substances from parent cells, facilitating cell-to-cell communication, and emerging as promising targets for the development of diagnostic, prognostic, and therapeutic agents. More diverse contents encapsulated within ApoEVs includes but is not limited to proteins, lipids, RNA, and DNA molecules 20. Tyukavin, A. I. *et al.* found that ApoEVs from cardiomyocytes enhance the proliferation and differentiation of resident stem cells through the transportation of specific miRNAs 36 and also mitigate myocardial infarction 18. ApoEVs have been reported to activate the Wnt/β-catenin pathway, promoting the proliferation, osteogenic differentiation, and lipogenic differentiation of BMSCs 37. In this study, we focused on the proteins that are increasingly expressed in ApoEVs compared to live BMSCs through bioinformatical analysis. Following gain-and loss-of-function assay confirmed that Ras is indispensable for ApoEVs to promote bone formation. However, since ApoEVs contains versatile bioactive proteins, further studies are necessary to find other important proteins in ApoEVs for disease treatment.

Ras proteins are classical members of small GTPases that function as molecular switches by alternating between inactive GDP-bound and active GTP-bound states. Anta, B. *et al.* documented the specific activation of H-Ras and N-Ras in cellular endomembranes during the process of apoptosis 38. In this study, Ras from apoptotic BMSCs were encapsulated into the ApoEVs, subsequently activating the Ras signal pathway in recipient BMSCs. Notably, Ras protein is an expression product of the oncogene c-Ras. The abnormal activation of Ras genes constitutes oncogenes, and the expression product Ras protein undergoes conformational changes 5 and functional changes, resulting in unanticipated uncontrolled proliferation 39, 40. However, in this article, ApoEVs treatment did not cause uncontrolled proliferation of BMSCs, because ApoEVs do not cause conformational changes of Ras protein in receptor cells but only increase the number of Ras, activating downstream pathways.

The Ras/Raf1/Mek/Erk pathway stands out as one of the most thoroughly characterized signal transduction pathways in cell biology. The pathway serves the pivotal function of transducing signals from the extracellular milieu to the cell nucleus where specific genes are activated to regulate processes such as cell death, cell migration, division, or differentiation 22, 23, 41. The Ras/Raf1/Mek/Erk pathway is implicated in wound healing and tissue repair 42, 43. Our data indicated that activation of the Ras/Raf1/Mek/Erk signaling pathway plays a critical role in the regulation of proliferation and osteogenic differentiation of BMSCs *in vitro* and ameliorate osteoporosis *in vivo*.

MSCT faces numerous challenges, including disparities in quantity and quality, instability in purity and properties, fragility in storage and transportation, susceptibility to pathogenic infections, and unanticipated differentiation *in vivo* 44, 45. Recently, a new concept of 'cell-free' stem cell therapy based on a bioactive component of stem cells has emerged 46, which is considered to overcome the above challenges of MSCT 47. Here, we proposed that ApoEVs offer distinct advantages as potential substitutes for therapeutic stem cells. The utilization of ApoEVs circumvents the metastasis risks associated with live cells, which may undergo mutations or damage DNA, thereby averting the potential for tumor development. Moreover, the smaller size of ApoEVs enables easy circulation, overcoming the limitation faced by MSCs in crossing the first capillary bed and enhancing treatment efficiency. The simplified extraction and purification process of ApoEVs obviates the need for laborious cell production methods, ensuring ease of quality control. Compared with other EVs (exosomes, micro-vesicles), ApoEVs are more suitable for large-scale clinical use due to their high yield and simple extraction scheme. In summary, ApoEVs, as a novel form of 'cell-free' stem cell therapy, offer a compelling solution by addressing ethical, tumorigenic, and immunogenic concerns associated with traditional MSCT.

## Materials and Methods

### Animal and ethics

All the animal experiments were approved by the Institutional Animal Care and Use Committee (IACUC) at West China Hospital of Stomatology, Sichuan University (WCHSIRB-D-2023-674). All the animals were purchased from Dossy Experimental Animal Co. Ltd. (Chengdu, China) and housed under standard pathogen-free conditions. The mice and rats were free to access food and water with ample sleep at 12 h alternation of day and night. 8-week male C57BL/6 mice and 4-week male SD rats were used to isolate BMSCs for *in vitro* experiments. GFP-C57BL/6 mice were used to isolate GFP-BMSCs. 8-week female C57BL/6 mice were used to set the OVX model.

### Reagents and chemicals

Staurosporine (#9953), phalloidin (#8953), anti-ERK1/2 antibody (#4695), and anti-p-ERK1/2 antibody (#4370) were purchased from Cell Signaling Technology (Danvers, Ma, USA). Annexin V (556420) and propidium iodide (PI) (556463) were purchased from BD Pharmingen (San Jose, CA, USA). BCA Protein Assay Kit (KGP902) and CCK-8 kit (KGA317s-1000) were purchased from KeyGEN BioTECH (Jiangsu, China). DAPI was purchased from Solarbio (Beijing, China). Anti-histone 3 antibody (PA5-16183) was purchased from Thermo (Carlsbad, CA, USA). CD63 (510953), β-actin (200068-8F10), cleaved-Caspase3 (380169), and Gapdh (200306) were purchased from Zen Bioscience. Anti-osteopontin antibody (ab63856), anti-Ki67 antibody- Proliferation marker (Ki67, ab15580), anti-Ras antibody (ab52939), and anti-Raf1 antibody (ab137435) were purchased from Abcam (Cambridge, UK). VybrantTM Dio Cell-Labeling Solution (V22886) was purchased from Life Technologies (New York, USA). Calcein (#C0875) was purchased from Sigma-Aldrich (St. Louis, Missouri, USA). OCN (sc-390877) and Nestin (sc-21248) were purchased from Santa Cruz (Dallas, U.S.A.).

### Cell culture

BMSCs were isolated from femurs and tibias of 8-week male C57BL/6 mice and 4-week male SD rats as previously described 48, 49. The hindlimbs were removed, and then BMSCs were separated by flushing marrow cavities and cultured at a density of 1.5×10^6^ cells per 10 cm culture dish (Corning, NY, USA) in α-modified Eagle's medium (α- MEM; HyClone), 10% fetal bovine serum (FBS; Gibco), 100 U/mL penicillin, and 100 U/mL streptomycin. BMSCs were cultured at 37 °C in 5% CO_2_ for 10 days. The medium was replaced every 3 days. Cells were digested and passaged twice for further experimental use by using 0.25% trypsin (Invitrogen, USA) at 80-90% confluence.

Osteoclastic medium (OCM) contained 10% FBS, 50 ng/mL MCS-F(R&D), and 100 ng/mL RANKL (R&D Systems, 462-TEC-010).

### Formation of GFP-bone marrow mesenchymal stem cell sheets

GFP- BMSCs isolated from GFP- C57BL/6 mice (purchased from Dossy Experimental Animal Co. Ltd.) were used to prepare the cell sheets. The extraction and cultivation methods are similar to normal BMSCs from normal C57BL/6 mice. 1.0×10^6^ of GFP-BMSCs at passage number 1 were sub-cultured in 10 cm dishes and cultured in a complete medium containing 20 μg/mL vitamin C. After culturing for 10 days, cells on the edge of dishes were wrapped, indicating the cell sheets had formed. The prepared GFP-BMSCs sheets were carefully clamped with tweezers to transplant into the femoral defect area and removed sheets at 2 days to detect the apoptotic rate of implanted cell sheets.

### Isolation of ApoEVs

ApoEVs were isolated using sequential centrifugation followed by a standard literature protocol 20, 50, 51. Briefly, after 500 nM staurosporine (STS) inducing apoptosis, the supernatants were centrifuged at 800× g for 10 min to remove cell debris. The ApoEVs-rich supernatant was centrifuged at 16000× g for 30 min to pellet the ApoEVs. The pellet was suspended in ice-cold PBS and was centrifuged at 16000× g for 30 min again to remove the contaminated protein. All centrifuge processes were performed at 4 °C to prevent the inactivation of ApoEVs. The purified ApoEVs were resuspended in 100 μL ice-cold PBS and stored at -80 °C immediately for future analysis.

### Characterization of ApoEVs

The morphology of ApoEVs was observed by Transmission Electron Microscopy (TEM). A total of 10 μL purified ApoEVs were placed on formvar- carbon-coated copper grids. After allowing ApoEVs attached to grids for 10 min, the liquid was removed from the edge of the grids by filter paper. ApoEVs on the grids were stained with 2% uranyl acetate for 10 min. Grids were washed with distilled deionized water for 10 min and then air-dried. The round shaped ApoEVs with a bilayer membrane were imaged by TEM at 80 kV. As for the ApoEVs ultrathin section, the samples were pre-fixed with 3% glutaraldehyde and then fixed with 1% osmium tetroxide. The sample is then dehydrated with acetone, embedded in epoxy resin, and prepared into ultrathin sections with a thickness of about 50 nm. They were stained with uranium acetate and lead citrate at room temperature and observed by TEM (Jem-1400plus).

The expression of Gapdh, histone 3, cleaved-caspase 3, and CD63 in ApoEVs was detected by Western blot. ApoEVs were stained with Annexin V-FITC. Flow Cytometry (Attune® NxT, ThermoFisher, CA, USA) analyzes the proportion of fluorescent intensity. 1 μm- and 4 μm-size calibration beads were used to gate ApoEVs. The average diameters of ApoEVs were detected by dynamic light scattering (DLS) using Zetasizer Nano ZSE (Anton Paar Litesizer 500). ApoEVs were quantified using the BCA Protein Assay Kit before use. 10 μg/mL ApoEVs were used as a work concentration in the following experiments.

### LC-MS/MS analysis

The ApoEVs and BMSCs samples were subjected to chemical treatment, tryptic digestion, and liquid chromatography (LC)-electrospray ionization (ESI) tandem mass-spectroscopy (MS/MS) analysis. LC-MS/MS analysis was performed on a timsTOF Pro mass spectrometry (Bruker) that was coupled to Nanoelute (Bruker). The peptides were loaded onto a C18-reversed phase analytical column (Thermo Scientific Easy Column, 25 cm long, 75 μm inner diameter, 1.9 μm resin) in 95% buffer A (0.1% Formic acid in water) and separated with a linear gradient of buffer B (99.9% acetonitrile and 0.1% Formic acid) at a flow rate of 300 nl/min. The mass spectrometer was operated in positive ion mode. The electrospray voltage applied was 1.5 kV. Precursors and fragments were analyzed at the TOF detector over a mass range of m/z 100-1700. The timsTOF Pro was operated in parallel accumulation serial fragmentation (PASEF) mode, PASEF mode data collection was performed based on the following parameters: Ion mobility coefficient (1/K0) value was set from 0.6 to 1.6 Vs cm^2^; 1 MS and 10 MS/MS PASEF scans. Active exclusion was enabled with a release time of 24 s.

### GO annotation and KEGG annotation

The protein sequences of the selected differentially expressed proteins were locally searched using the NCBI BLAST^+^ client software and InterProScan to find homologue sequences, then gene ontology (GO) terms were mapped, and sequences were annotated using the software program Blast2GO. The GO annotation results were plotted by R scripts.

Following annotation steps, the studied proteins were blasted against the online Kyoto Encyclopedia of Genes and Genomes (KEGG) database (http://geneontology.org/) to retrieve their KEGG orthology identifications and were subsequently mapped to pathways in KEGG.

### Protein-protein interaction analysis

The protein-protein interaction (PPI) information of the studied proteins was retrieved from IntAct molecular interaction database (http://www.ebi.ac.uk/intact/) by their gene symbols or STRING software (http://string-db.org/). The results were downloaded in the XGMML format and imported into Cytoscape software (http://www.cytoscape.org/, version 3.2.1) to visualize and further analyze functional protein-protein interaction networks. Furthermore, the degree of each protein was calculated to evaluate the importance of the protein in the PPI network.

### *In vivo* imaging system

DIO-labeled ApoEVs or BMSCs were used for near-infrared fluorescence (NIRF) *in vivo* distribution imaging. After tail vein injection for 1 h, 6 h, 24 h, and 48 h, the mice were anesthetized and sacrificed. The heart, mandibular, lungs, liver, spleen, kidneys, and upper/lower legs with or without surface muscle were dissected. A fluorescence tomography *in vivo* imaging system (PerkinElmer, FMT4000) was employed to capture distribution images.

### ApoEVs labeling and cellular engulfment assay

ApoEVs were labeled with 10 μL membrane-labeling dye PKH67 (MKCK0731, Sigma, Massachusetts, USA) at 37 °C for 20 min according to the manufacturer's protocol. BMSCs were cultured on confocal dishes and treated with 4×10^6^ PKH67-labeled ApoEVs per 1×10^6^ BMSCs for 24 h. Cells were fixed in 4% paraformaldehyde, stained with phalloidin, and DAPI and imaged by confocal microscopy (Olympus FV1000, Japan).

### Cell proliferation assay

BMSCs at a density of 2×10^3^ / well were inoculated in 96-well plates (Corning, NY, USA) and co-cultured with PBS or ApoEVs. At the designated times (1, 2, 3, 4, and 5 days), cell proliferation ability was assessed using the cell counting kit‐8 (CCK8) assay according to the manufacturer's instructions. The absorbance value at 450 nm was detected to analyze cell proliferation viability.

### Wound healing assay

For the wound healing assay, BMSCs were seeded on 24-well plates (Corning, NY, USA). At 90% confluence, scratch wounds were created by pipette tips scratching the cell monolayer. BMSCs were continuously incubated with PBS or ApoEVs for 24 h. 4% paraformaldehyde-fixed for 30 min and 0.05% crystal violet stained. Pictures of the scratch were taken under a microscope and the motility of cells was compared by the degree of confluence from one side of the scratch to the other.

### Boyden chamber assay

For the cell migration assay, 1×10^6^ BMSCs were plated into the upper chamber of a 24-well 8 μm pore size transwell device (Corning Incorporated, Corning, NY, USA). ApoEVs were added to the lower chamber as a chemoattractant, while PBS was added as a control group. 24 h after incubation at 37 ℃, cells on the top surface of the chamber were removed and cells on the underside of the chamber were fixed in 4% paraformaldehyde for 30 min, stained with 0.05% crystal violet, and air-dried. Migrated cells were quantified under an inverted light microscope (Olympus, Japan).

### Osteogenic differentiation assay and TRAP staining

5×10^5^ BMSCs were seeded into a 24-well plate per well. When cells reached 100% confluence, the basal medium was changed into osteogenic induction medium: α-MEM containing 10% FBS, 1% penicillin/streptomycin, 2 mM β-glycerophosphate (D301908, Aladdin, Shanghai, China), 100 μM ascorbic acid 2-phosphate (A103534, Aladdin), and 10 nM dexamethasone (D137736, Aladdin). BMSCs were treated with PBS or ApoEVs. To detect mineral nodule formation, after culturing in the osteogenic medium for 14 days, BMSCs were fixed with 4% PFA for 30 min at room temperature, and then Alizarin red staining was performed. Alizarin red-positive rate was analyzed by detecting absorbance (OD at 562 nm) after extracting by 10% Cetylpyridinium chloride (A600106-0100, Sangon Biotech, Shanghai, China).

Trap staining was carried out to examine the osteoclast in tissue. Briefly, the slices are dewaxed in xylene, hydrated with distilled water, drip-filtered TRAP working solution to cover the tissue, and react at the 37 ℃ in dark for 30 min. Pour out the incubation solution, wash with water and counterstain with hematoxylin for visualization and quantification.

### qRT-PCR and western blot

The mRNA of BMSCs in different groups was extracted. The RNA-easy Isolation Reagent (Vazyme, China) was used to extract the total RNA according to the instructions. The Revert Aid First Strand cDNA Synthesis Kit (Vazyme, China) was used to reverse-transcribe 1 μg mRNA to cDNA. Relative-quantitative PCR analyses were performed using a real-time PCR System Quant Studio 6 Flex system (Applied Biosystems, USA). All the primer sequences used in this experiment can be seen in Table S1.

Purified ApoEVs and BMSCs were dissolved in RIPA Lysis Buffer (KGP702-100, KeyGEN, China). After being quantified by BCA assay, the proteins were loaded on sodium dodecyl sulfate-polyacrylamide (SDS) gels and transferred to polyvinylidene fluoride (PVDF) membranes (Millipore, USA). Then, the membranes were blocked with 5% BSA for 2 h at room temperature and incubated with primary antibodies overnight at 4 °C. Finally, the membranes were incubated with peroxidase-conjugated secondary antibodies for 1 h at room temperature. The protein bands were detected by the imaging system and quantified by ImageJ software.

### Animal experiments

The main contents of animal experiments were approved by the Ethical Review Board at the West China Hospital of Stomatology, Sichuan University, based on the Experimental Animal Management Ordinance (National Science and Technology Committee of the People's Republic of China, 1988). Four-week-old male mice were anesthetized and made the femoral defect model with a size of 1 mm. GFP-BMSCs sheets were transplanted into the defect area. Two-month-old C57BL/6 female mice were anesthetized and subjected to bilateral ovariectomy (OVX). One week after ovary removal surgery, 2×10^5^ BMSCs or 100 μg of ApoEVs from 2×10^5^ BMSCs or an equal volume of PBS were injected once per week by the tail vein. After 2 months of treatment, femurs were collected and fixed in 4% paraformaldehyde for further analyses.

### Micro-computed tomography (μCT) analysis

Collect femurs from mice after administration, 4% paraformaldehyde was used to fix for 24 h, then placed vertically in a 19 mm scanning tube to scan using a Skyscan 1176 (Skyscan). All the scan parameters were as follows: the scanner voltage was 70 kV and the current was 200 μA, with the resolution set to 9 μm per pixel. After the scanning, the region of interest (ROI) selected for analysis and 3D reconstruction was 1.35 mm in thickness from 0.45mm below the growth plate. Data analysis software (CTAn) was used to determine the bone volume (BV), trabecular bone volume per tissue volume (BV/TV), trabecular number (Tb.N), trabecular separation (Tb.Sp), trabecular thickness (Tb.Th), and bone mineral density (BMD). The image reconstruction software was used for further 3D reconstruction.

### Calcein deposition experiment

The calcein deposition experiment was used to explore the new bone deposition conducted as the previous experiments with a modification. In brief, the mice were injected with 10% calcein at the dose of 15 mg/Kg 10 days and 3 days before sacrifice. The tibias were collected, fixed, dehydrated respectively, and immersed in the plastic solution for 30 days. After embedding with methyl methacrylate, the bones were sliced into 30 μm thickness sections. The fluorescence images of calcein distribution were captured under a confocal laser scanning microscope (CLSM, Olympus, Japan). The distance between the two green lines was measured by using Image J software, and the quantitative analysis of mineral apposition rate (MAR) was performed by the formula (the distances/7 days) in three randomly chosen fields.

### Histochemistry detection of bone tissue

Immunofluorescence (IF) staining was carried out to examine the osteogenic marker in bone tissue. Briefly, the slices are dewaxed in xylene, hydrated with distilled water, and treated with 1% TritonX-100 to increase permeability. Primary antibodies of OPN (1:200, Abcam, USA) were incubated at 4 °C for overnight. The secondary antibody Alexa Fluor 488 goat anti-mice (1:500, Invitrogen, USA) was incubated at 37 °C for 1 h. DAPI (1:1000, Solarbio, China) was used to stain the nuclei. The fluorescence images were captured under a confocal laser scanning microscope (CLSM, Olympus, Japan). The protein accumulation of OPN was examined by counting the number of green (the positive area within cells) within the linear surface area divided by the total number of pixels. All the parameters were calculated by three randomly chosen fields.

As for immunohistochemistry (IHC) staining, paraffin-embedded bone sections were dewaxed and hydrated. To block endogenous peroxidase activity, sections were incubated in 3% hydrogen peroxide for 15 min at room temperature. After washing with PBS, the slides were blocked with blocking buffer for 1 h and incubated with anti-OPN (1:200) antibody overnight at 4 °C. After washing, slides were incubated with biotinylated secondary antibody (1:200) for 1 h at 37 °C. After incubating with HRP- streptavidin complex for 30 min, slides were developed until light brown staining was visible with the DAB chromogen kit (GK600710, Gene Tech).

### siRNA knockdown

siRNAs for KRas were used to treat BMSCs according to the manufacturer's instructions (HangBio, Shanghai). Non-targeting control siRNAs (si-NC) were used as negative controls. MAPK1-targeting control siRNA (si-PC) was used as a positive control. Successful transfection was verified by qRT-PCR and western blot. All the primer sequences can be seen in Table S2.

### Lentivirus for gene overexpression

EGFP-KRas fusion protein expression lentivirus (HangBio, Shanghai) was used to overexpress the Ras of BMSCs according to the manufacturer's instructions. Empty lentivirus with the same backbone was used as a control. The ApoEVs overexpressed by Ras were generated from transfected BMSCs, which were assessed by qRT-PCR and western blot. The carrier map can be found in the Figure S8. ZsGreen protein expression lentivirus (HangBio, Shanghai) was used to overexpress the ZsGreen of BMSCs according to the manufacturer's instructions.

### Statistical analysis

All data are representative of at least three experiments of similar results performed in triplicate unless otherwise indicated. Data are expressed as mean±SD. Comparisons between two groups were analyzed using independent unpaired two-tailed Student's t-tests, and comparisons between more than two groups were analyzed using one-way ANOVA with the Bonferroni adjustment. P values less than 0.05 were considered statistically significant.

## Supplementary Material

Supplementary figures and tables.

## Abbreviations

MSCs: Mesenchymal stromal cells
BMSCs: bone marrow mesenchymal stem cells
ApoEVs: apoptotic extracellular vesicles
ApoBDs: apoptotic bodies
ApoMVs: apoptotic cell-derived microvesicles
OVX: ovariectomy
IVIS: *In Vivo* Imaging System
NIRF: near-infrared fluorescence
FCM: Flow Cytometry
OPN: osteopontin
DEPs: differentially expressed proteins
IHC: Immunohistochemistry
STS: staurosporine
TEM: Transmission Electron Microscopy
DLS: dynamic light scattering
