# Supplementary material for: Extracellular vesicles from apoptotic BMSCs ameliorate osteoporosis via transporting regenerative signals
Source: Theranostics. 2024 Jun 3;14(9):3583–602. doi: 10.7150/thno.96174 (PMC11209718; doi:10.7150/thno.96174)
Supplement: Supplementary file 1 — Supplementary figures and tables. [file thnov14p3583s1.pdf]

Supplementary Information

Extracellular vesicles from apoptotic BMSCs ameliorate osteoporosis via transporting regenerative signals

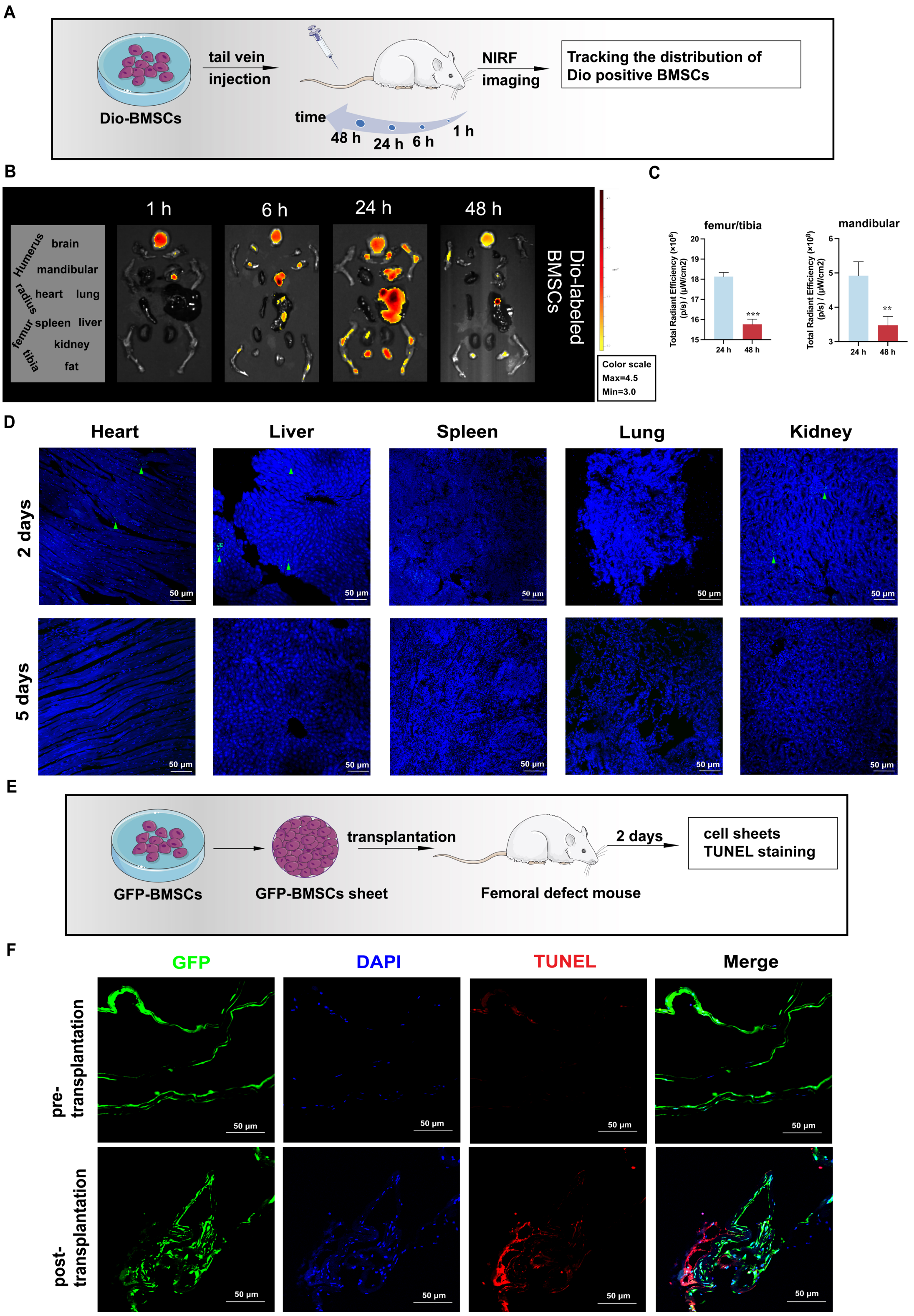

Figure S1. BMSCs undergo apoptosis post-transplantation. (**A**) Schematic diagram outlining the experimental design for tracking the fate of BMSCs post-transplantation. (**B**) Fluorescence images depicting major organs at 1 h, 6 h, 24 h, and 48 h after injection of Dio-labeled BMSCs. (**C**) Analysis Fluorescence intensity in femur/tibia and mandibula 24 h and 48 h after injection of Dio-labeled BMSCs. (**D**) Confocal assay of ZsGreen-BMSCs distribution in various organs (heart, liver, spleen, lung, kidney) after 2 and 5 days of tail vein injection of ZsGreen-BMSCs. Scale bar: 50 μm. (**E**) Schematic diagram illustrating the experimental design for detecting the apoptosis of GFP-BMSCs post-transplantation into femoral defect mice. (**F**) TUNEL staining showing GFP^{+} BMSCs undergo apoptosis. Scale bar: 50 μm. Data are means ± SEMs. Statistical significance was determined by a two-tailed paired t-test in (C). **P < 0.01, ***P < 0.001.

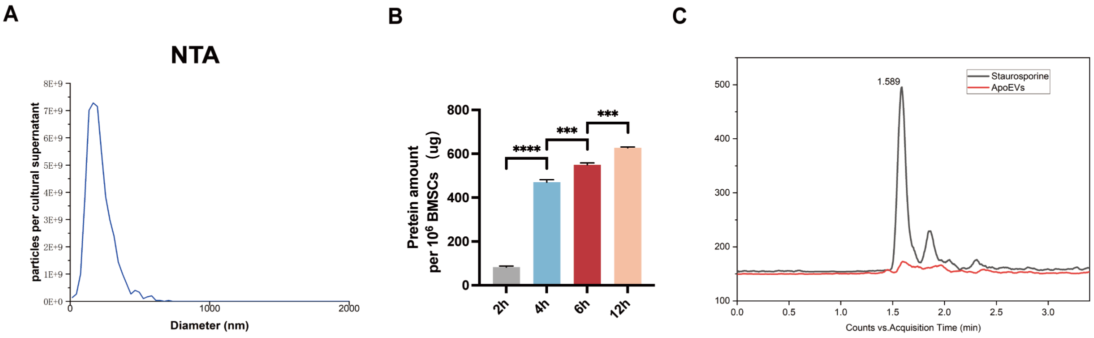

Figure S2. Identifying of ApoEVs. (A) NTA showed the diameter range and nanoparticles of ApoMVs. **(B)** BCA detects the protein amount of ApoEVs from 1×10^{6} BMSCs after STS inducing apoptosis at different times. **(C)** HPLC-MS/MS showed no STS residue in the extracted ApoEVs compared to the positive control group containing only STS drugs. ***P < 0.001.

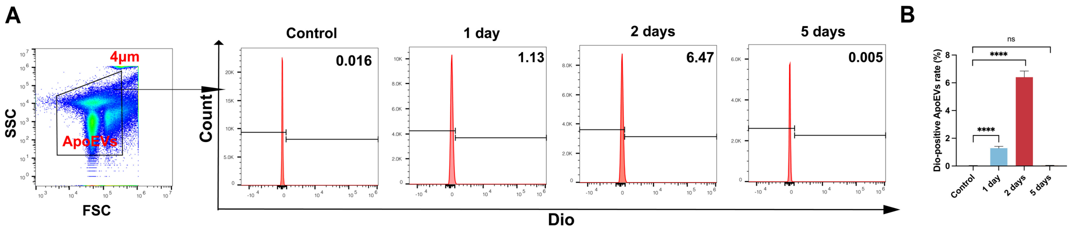

Figure S3. ApoEVs production at different times after tail vein injection of Dio-BMSCs. (A-B) The Dio-labeled ApoEVs were detected by FCM after Dio-BMSCs were injected at different times. Not significant (ns) = P > 0.05, ****P < 0.0001.

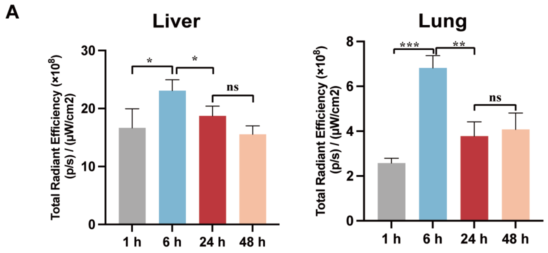

Figure S4. ApoEVs accumulate in liver and lung. (**A**) Fluorescence intensity analysis of the liver and lungs at 1 h, 6 h, 24 h, and 48 h after injection of Dio-labeled ApoEVs. Data are means ± SEMs. Statistical significance was determined by a two-tailed paired t-test in (A). Not significant (ns) = P > 0.05, *P < 0.05, **P < 0.01, ***P < 0.001.

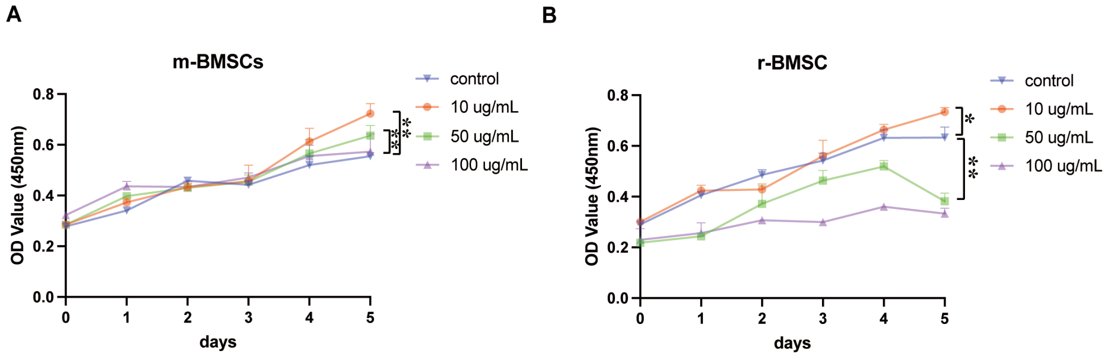

Figure S5. ApoEVs promoted the proliferation of BMSCs in low concentrations. (A) The effect of 10 μg/mL 50μg/mL and 100 μg/mL ApoEVs on the proliferation ability of m-BMCSs. **(B)** The effect of 10 μg/mL, 50 μg/mL, and 100 μg/mL ApoEVs on the proliferation ability of r-BMCSs. *P < 0.05, **P < 0.01.

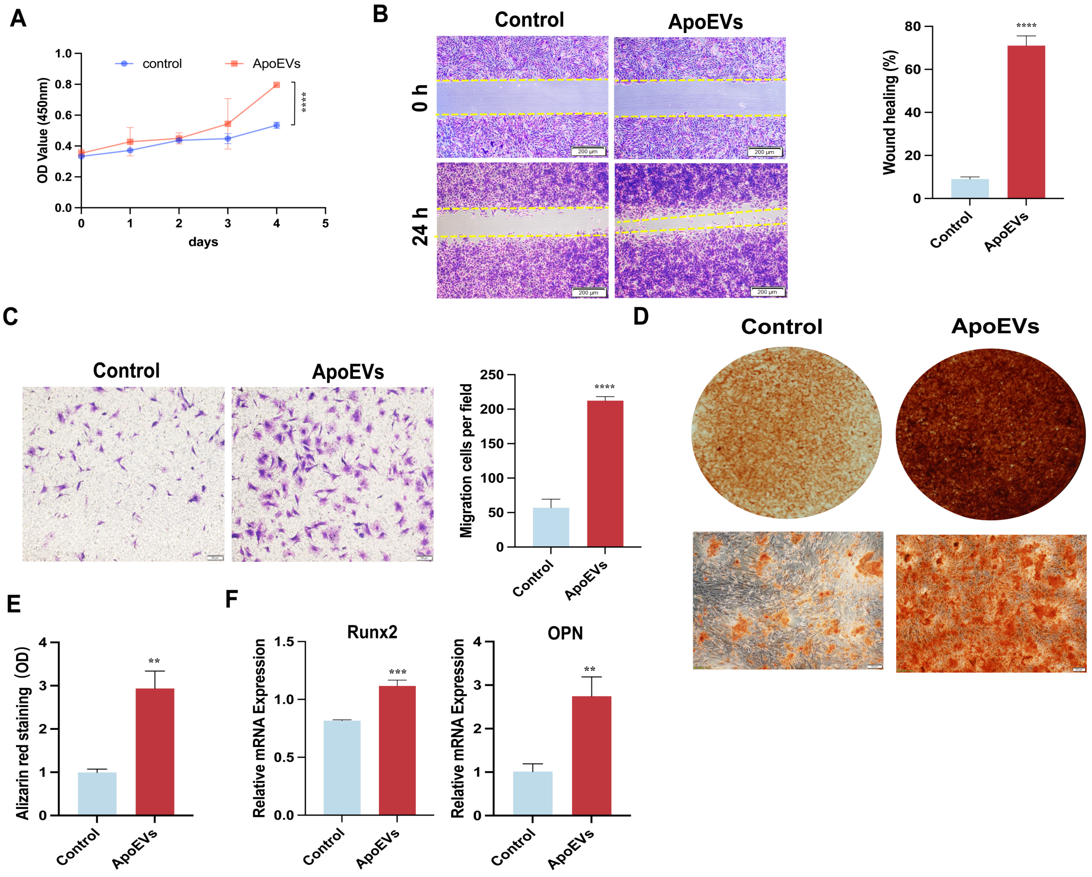

Figure S6. The effect of ApoEVs on the proliferation, migration, and osteogenic differentiation of r-BMSCs. (**A**) The effect of ApoEVs on the proliferation ability of BMSCs was measured by the CCK-8 assay. (**B**) Scratch healing assay and transwell assay (**C**) illustrate the effect of ApoEVs on the migration and invasion ability of BMSCs. Scale bar: 200 μm (B), and 100 μm (C). (**D-E**) ApoEVs increased the mineralized nodule formation of BMSCs during osteogenic induction, as assessed by alizarin red staining. Scale bar: 100 μm (**F**) ApoEVs from r-BMSCs promote the expression of Runx2 and OPN, as assessed by qRT-PCR. Data are means ± SEMs. Statistical significance was determined by a two-tailed paired t-test in (A, B, C, E, F). Not significant (ns) = P > 0.05, *P < 0.05, **P < 0.01, ***P < 0.001, ****P < 0.0001.

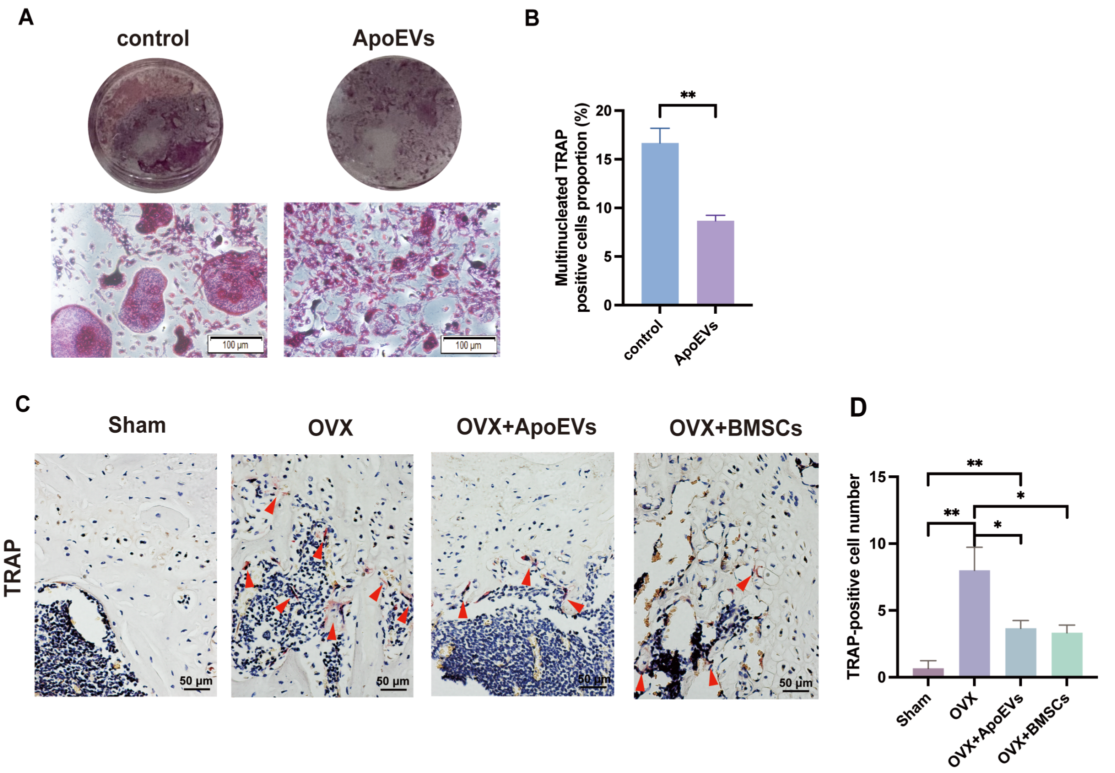

Figure S7. The effect of ApoEVs on osteoclasts. (A-B) Trap stain showed that ApoEVs inhibit osteoclast formation. Scale bar: 100 μm. **(C-D)** ApoEVs inhibit bone resorption as assessed by Trap-stained femur slices. Scale bar: 50 μm. *P < 0.05, **P < 0.01.

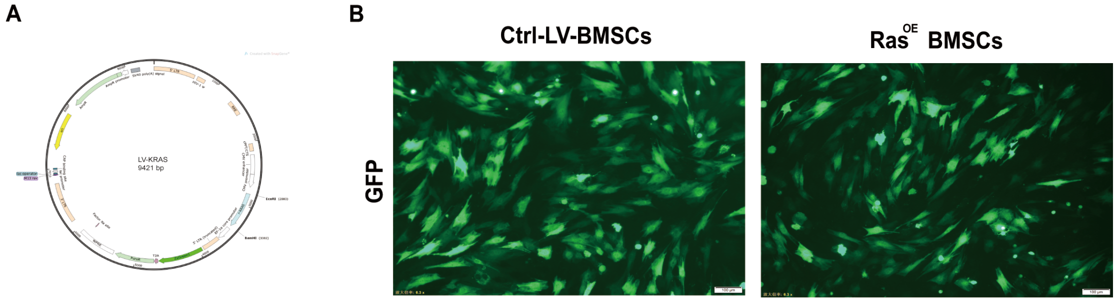

Figure S8. Ras lentivirus successfully transfected into BMSCs. (**A**) Schematic diagram outlining the gene targeting strategy for generating Ras overexpression in lentivirus. (**B**) GFP-Ras lentivirus successfully transfected into BMSCs. Scale bar: 100 μm.

Table S1. The sequences of the qRT-PCR primers.

Gene	Forward primer sequence 5′→3'	Reverse primer sequence 5′→3'
**Mouse**		
ALP	TTGTGCCAGAGAAAGAGA	GTTTCAGGGCATTTTTCAAGGT
RUNX2	CCGCACGACAACCGCACCAT	CGCTCCGGCCCACAATCTC
OCN	CTGACAAAGCCTTCATGTCCAA	GCGCCGGAGTCTGTTCACTA

Ras	GCTCACCATCCAGTTCATCCAGTC	GGCTGCTCTGTCATCTATCACACAC
Raf1	CAGATGTGGCACGGAGCAACC	CTCGGACTGTAACTCCACACCTTG
MEK	TCATCTGGAGATCAAACCCGCAATC	CCATCGCTGTAGAACGCACCATAG
ERK1/2	CCACCTGTGAAGAATGAACT	CACCTTTCTCTGTCTTTATCGT
Ki67	CACAGAGAACAAAGGTGTGAAG	GGAGACTGCAGAGCTATTTTTG
cyclinD2	TGGGAAGTTTTGTTGGGTCA	TCCTTGTCCAGGTAATGCCA
CDK2	CCTGCTTATCAATGCAGAGGG	GTGCTGGGTACACACTAGGTG
Gapdh	AAGAAGGTGGTGAAGCAGGCATC	CGGCATCGAAGGTGGAAGAGTG
**Rat**		
RUNX2	TCATTTGCAsCTGGGTCACAT	TCTCAGCCATGTTTGTGCTC
OPN	AAGCGTGGAAACACACAGC	CTTTGGAACTCGCCTGACTG
Gapdh	TATGACTCTACCCACGGCAAG	TACTCAGCACCAGCATCACC

Table S2. The sequences of the siRNA primers.

**Gene**	**Forward primer sequence 5′→3'**	**Reverse primer sequence 5′→3'**
siRNA NC	UUCUCCGAACGUGUCACGUTT	ACGUGACACGUUCGGAGAATT
siRNA PC	CCCUCACAAGAGGAUUGAATT	UUCAAUCCUCUUGUGAGGGTT
siKRAS	CCAUUAUAGAGAACAAAUUTT	AAUUUGUUCUCUAUAAUGGTT
